# Assembly PCR synthesis of optimally designed, compact, multi-responsive promoters suited to gene therapy application

**DOI:** 10.1038/srep29388

**Published:** 2016-07-08

**Authors:** H. Mohamed, Y. Chernajovsky, D. Gould

**Affiliations:** 1 Queen Mary University of London, William Harvey Research Institute, Bone & Joint Research Unit, United Kingdom.

## Abstract

Gene therapy has the potential to provide innovative treatments for genetic and non-genetic diseases, with the ability to auto-regulate expression levels of therapeutic molecules so that they are produced locally and in direct response to disease activity. Generating disease responsive gene therapy vectors requires knowledge of the activation profile of transcription factors (TFs) during active disease, in order to assemble binding sites for these TFs into synthetic promoters, which can be appropriately activated by the disease process. In this study, we optimised a PCR random assembly approach to generate promoters with optimal spacing between TF binding sites (TFBSs) and their distance from the TATA box. In promoters with optimal spacing, it was possible to demonstrate activation by individual transcription pathways and either additive or synergistic promoter activation when transfected cells were treated with combined stimuli. The kinetics and sensitivity of promoter activation was further explored in transduced cells and when lentivirus was directly delivered to mouse paws a synthetic promoter demonstrated excellent activation by real-time imaging in response to local inflammation.

After 30 years of endeavour, there are a growing number of gene therapy successes in both monogenetic^1^,^2^,^3^,^4^,^5^,^6^ and polygenetic conditions^7^,^8^,^9^,^10^. With each success, the potential for gene therapy expands and the array of gene therapy tools increases. To date these strategies have involved either *ex vivo* engineering of stem cells which are re-introduced to patients or *in vivo* direct delivery of vectors to patients. There is however, further scope to increase the specificity of gene therapy approaches to achieve more advanced and innovative treatments. One approach is the development of ‘physiologically responsive’ gene therapy. This term was coined by Varley and Munford in 1998^11^ to describe the use of endogenous promoters that drive expression of therapeutic proteins in direct response to disease stimuli. The authors demonstrated the inflammation responsiveness of promoters for the acute phase proteins; complement factor 3 (C3) and serum amyloid A3 (SAA3), following delivery to the mouse liver with adenoviral vectors^12^.

Several responsive endogenous promoters have been utilised in experimental gene therapy studies^13^,^14^,^15^, as well as hybrid promoters such as IL-1E/IL-6P which is composed of an enhancer from the IL-1 promoter (−3690/−2720) and the proximal IL-6 promoter (−163/+12)^16^,^17^,^18^. However, the size of endogenous and hybrid promoters is usually in excess of 1 Kb which can preclude their application in vectors where there is limited capacity. Endogenous promoters can also harbour binding sites for TFs not relevant to the disease process which could lead to inappropriate promoter induction and unwarranted activation profiles that do not mirror the course of the disease for which they are intended.

An alternative approach is to generate smaller synthetic promoters in which *cis*-acting recognition motifs for a particular TF are cloned upstream from a core promoter. Promoters of this type, such as NF-κB and HIF-1α responsive promoters have been explored in experimental gene therapy applications^19^,^20^,^21^,^22^,^23^,^24^,^25^,^26^, however, mono-responsive synthetic promoters are unlikely to have an activation profile that mirrors the course of a disease in its entirety, therefore, it would be logical to utilise promoters that are activated by several TFs relevant to the disease process under study. In a previous study different TFBSs were combined to create a multi-responsive synthetic promoter, but additive induction was not observed with combined stimulation^27^.

In this study, we aimed to generate compact and optimally functioning multi-responsive promoters with low basal activity in the absence of stimulation and dose dependent additive induction with increasing stimulation. We utilised a PCR assembly method to generate promoters and explored the optimal spatial arrangement of TFBSs relative to each other and to the TATA box within the core promoter. We then applied this optimal template to the generation of promoters harbouring TFBSs for three different TFs and demonstrate that with randomly assembled promoters we can yield synthetic promoters with the induction characteristics we required. Induction of these promoters was fully characterised *in vitro* and when tested *in vivo* they displayed appropriate activation in a disease model.

## Results

### Activity of mono-responsive promoters in cells exposed to multiple stimulation

**Figure 1:**
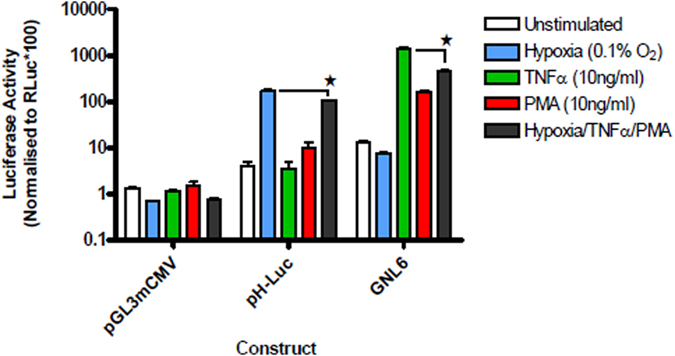
Compromised function of mono-responsive promoters upon multiple stimulation. 293T cells were transfected with constructs and were either unstimulated (white bars) or stimulated with hypoxia (0.1% O_2_ – HIF-1α) (blue bars), TNFα (10 ng/ml – NF-κB) (green bars), PMA (10 ng/ml – AP-1) (red bars) or their combination (grey bars)(the same colour for stimulation is used in subsequent figures). After 24 hours luciferase activity in cell lysates was quantified and mean values of triplicate normalised readings plotted with vertical lines representing the SEM. The responsiveness of each promoter to stimulation was also analysed by 1 way ANOVA (within each promoter) which showed significant differences. A post-hoc Šidak test was then performed to determine when the activity with combined stimulation (grey bars) was significantly (p < 0.05) lower than the highest activation caused by an individual stimuli this is indicated by ★.

Disease pathology is complex with the activation and involvement of multiple cell signalling pathways at different stages of disease progression. As such, profiling of disease responsive promoters applicable to gene therapy application should not be limited to activation of their responsive pathway but should also be monitored when other disease related pathways are activated. We therefore examined the activation of previously reported mono-responsive promoters in transfected cells. These studies showed that a hypoxic responsive promoter in the plasmid pH-Luc^23^ was activated by hypoxia (0.1%) with a 43 fold increase in luciferase activity but when transfected cells were stimulated with hypoxia, TNFα and PMA in combination, the induction was significantly lower at 27 fold. Similarly, the NF-κB promoter in pGNL6^20^ was induced with TNFα by 107 fold, but the same combined stimulation only resulted in an induction of 34 fold (Fig. 1). These findings illustrate that either mono-responsive promoters need to be designed carefully so that they retain full activity even when other transcriptional pathways are activated or alternatively, multi-responsiveness should be built into promoters so that other relevant transcription factors contribute to their activation.

### Influence of close TFBS alignment in composite promoters on basal promoter activity and induction

**Figure 2:**
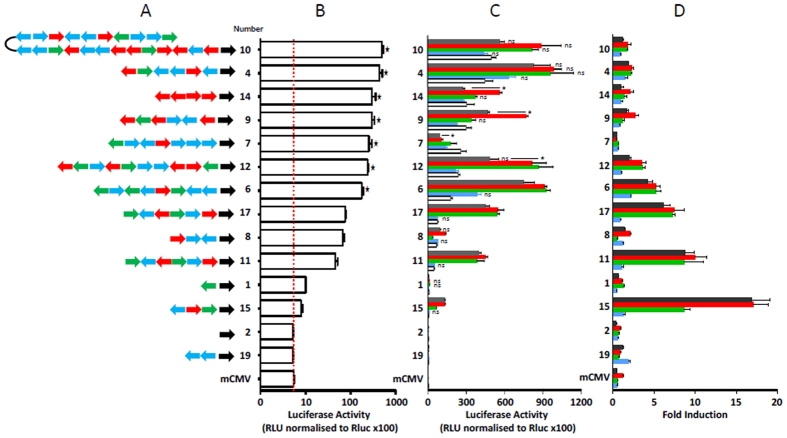
Structure and function of 4bp composite synthetic promoters. Compact randomly assembled 4bp synthetic promoter sequences are schematically represented in (**A**) (TFBS: AP-1 (red), NF-κB (green), HRE (blue) the arrow direction indicates the orientation of TFBS and the black arrow represents the mCMV promoter). Basal promoter activity was assessed in transfected non-stimulated 293T cells and is illustrated in (**B**), comparison of basal expression of all promoters with the mCMV group was performed by 1 way ANOVA which showed significant differences. A post-hoc Tukey test was then performed to determine significant differences from the mCMV promoter and where p < 0.05 this is indicated by ★. (**C)** shows the luciferase activity upon stimulation of cells and these changes are expressed as fold changes from basal activity in (**D)**. The responsiveness of each promoter in C to stimulation was also analysed by 1 way ANOVA (within each promoter). A post-hoc Šidak test was then performed to determine the level of significance of mean values above the activity of the unstimulated promoter. Where the difference from unstimulated promoter activity was not significant (p > 0.05) this is indicated by ns. When the activity with combined stimulation (grey bars) was significantly (p < 0.05) lower than the highest activation caused by an individual stimuli this is indicated by ★.

Many previously reported synthetic regulated promoters have been assembled with TFBS closely aligned to each other^21^,^28^ so we explored this approach to generate multi-responsive synthetic promoters. Initial experiments were performed by combining binding sites for the TFs NF-κB, AP-1 and HIF-1α (with a 4 bp separation between each TFBS) upstream of the mCMV promoter and the firefly luciferase reporter gene, by random ligation of pre-annealed oligonucleotides (sequences detailed in Supplementary Table 1). The sequences of these promoters (n = 13) were determined (Supplementary Material Appendix) and are represented schematically (Fig. 2A). In transiently transfected 293T cells, the majority of the promoters exhibited high basal luciferase expression (average of 186.7 RLU) in comparison to the basal activity demonstrated by the mCMV core promoter within the negative control plasmid (5.4 RLU) (Fig. 2B). Promoters with few TFBS inserts displayed lower basal activity and generally, increasing the TFBS number resulted in higher basal activity. In terms of induction, the 4bp-composite promoters were generally responsive to each individual stimuli in accordance with their TFBS composition (Fig. 2C), however, due to their elevated basal activity, low fold inductions were generally observed (Fig. 2D). Promoter 15 displayed a combined induction in excess of 15 fold and this promoter consisted of a single TFBS for each transcription factor resulting in low basal activity. Despite being responsive to each stimuli, promoter 15 did not display additive induction following combined stimulation. The general lack of additive induction in these promoters may have been due to the close proximity of TFBSs which could have resulted in the steric hindrance of adjacent TF binding. We therefore explored whether the spatial arrangements of TFBSs could be optimised to reduce basal promoter activity and facilitate additive induction of composite promoters.

### Optimised construction of synthetic promoters by Assembly PCR

The Assembly PCR method is a two-step PCR approach (assembly and amplification) conventionally used to synthesise genes from multiple overlapping oligonucleotides^29^. Modifications to the Assembly PCR method described by Team Heidelberg (http://2009.igem.org/Team:Heidelberg/Project_Synthetic_promoters) led to the generation of libraries of single-responsive synthetic promoters with evenly spaced TFBSs. We further modified this approach to enable the assembly of synthetic promoters containing multimerised TFBSs cloned upstream of the mCMV promoter and the firefly luciferase gene (Supplementary Figure 1A).

The use of overlapping annealing sequences devoid of any TFBSs is fundamental to the Assembly PCR method to ensure that the annealing sequences are transcriptionally silent and this was confirmed using the TRANSFAC database^30^. We then determined optimal conditions for Assembly PCR promoter construction by first exploring the influence of annealing sequence length on PCR product formation (sequences detailed in Supplementary Table 2). Hypoxic response element (HRE)-oligonucleotides with 10 bp, 15 bp and 20 bp annealing sequences were assembled and amplified using Assembly PCR and analysis of the resulting PCR products by gel electrophoresis confirmed the assembly and amplification of PCR products containing 15 bp and 20 bp annealing sequence. In contrast, the PCR products generated using the 10 bp annealing sequences were very small and indistinguishable from the amplification ‘stop’ primers (Supplementary Figure 1B and C) indicating inefficient PCR assembly, therefore, annealing sequences of 15 bp or 20 bp were used in subsequent reactions.

The ‘stop’ oligonucleotides serve three important functions in the Assembly PCR reaction; firstly, they truncate the PCR product to prevent infinite assembly, secondly, the incorporated 5′- and 3′-‘stop’ oligonucleotides in the assembled PCR product serve as primer binding sites during the subsequent amplification reaction and thirdly, they contain restriction enzyme sites for further cloning. Therefore, the efficient incorporation of the ‘stop’ oligonucleotides in the PCR product during the initial ×10 cycle assembly reaction improves the efficiency of the amplification reaction, restriction digestion and ligation reactions. We compared the use of ‘stop’ oligonucleotides over the concentration range of 16 nM to 260 nM, with equimolar forward and reverse-HRE oligonucleotides containing a 20 bp annealing sequence. As expected, increasing the ‘stop’ oligonucleotide concentration in the initial assembly reaction led to a reduction in the size of the assembled and amplified PCR products (Supplementary Figure 1D and E, respectively), indicating the efficient truncation of the assembled PCR products with higher concentrations of ‘stop’ oligonucleotides. In order to generate compact promoters, we selected a ‘stop’ oligonucleotide concentration of 210 nM for use in subsequent assembly reactions.

### Optimal spatial arrangement of  TFBSs relative to each other and to the TATA box for low basal promoter activity and maximal induction

**Figure 3:**
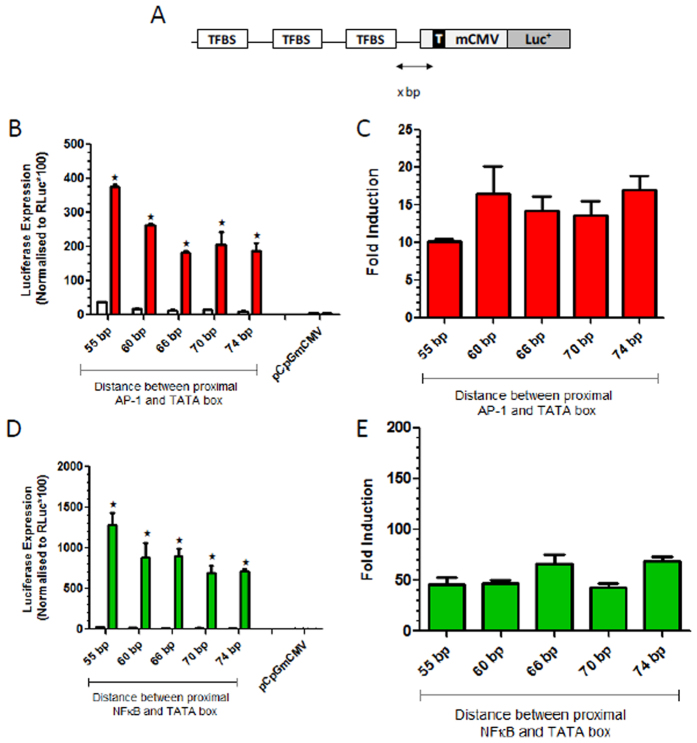
Optimisation of spacing between the TATA box and the proximal TFBS. The spacing between the TATA (T) box in the minimal CMV (mCMV) promoter and the proximal TFBS was altered as represented in (**A)** to examine the influence on regulated gene expression. The effect of increased spacing between the proximal AP-1 site and the TATA box was assessed in transfected 293T cells that are unstimulated (white bars) and stimulated with PMA (red bars) in B and as fold induction in (**C)**. Similar studies on spacing between the proximal NF-κB site and the TATA box in transfected 293T cells stimulated with TNFα (green bars) are shown in (**D,E)**. Data in (**B,D)** was analysed by 2 way ANOVA to take into account the effect of the change in sequence length and the effect of stimulation upon the promoters. This analysis revealed that both factors had significant effects on promoter activity. A post-hoc Šidak test was then performed to compare the stimulated and non-stimulated activity of each promoter and significant differences of p < 0.05 are indicated by ★.

Next, we investigated the effect of spatial preferences of proximal AP-1 and NF-κB motifs relative to the TATA box within the synthetic promoters. PCR assembled promoters containing 8xAP-1 or 6xNF-κB motifs were cloned into the mCMV-Xbp vector which permitted systematic expansions over the range of 55 bp and 74 bp between the proximal TFBS and the TATA box (Fig. 3A). Interestingly, both AP-1 and NF-κB-responsive promoters with the proximal TFBS located closest to the TATA box (55bp space) had the highest basal and highest induced luciferase gene expression levels and as the proximal TFBS was positioned further from the TATA box, the basal and induced gene expression levels declined (Fig. 3B,D, respectively). Due to the higher basal expression with 55 bp spacing from the TATA box, the fold induction was lower at this distance with both AP-1 and NF-κB TFBSs (Fig. 3C and E respectively). The 66bp distance from the TATA box gave lower basal expression than the 55 bp space when tested with NF-κB and AP-1 TFBSs and retained high induced luciferase expression, and was therefore utilised in all subsequent synthetic promoters.

**Figure 4:**
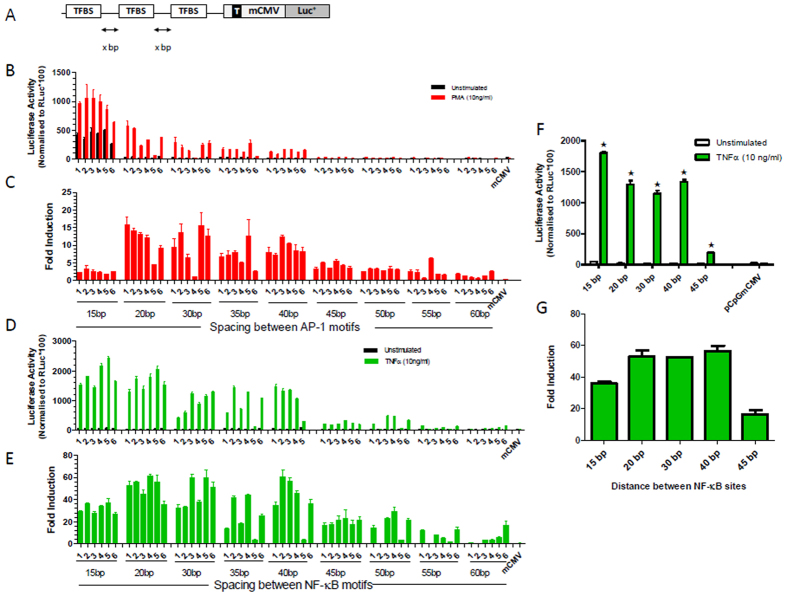
Optimisation of the spacing between TFBS in assembled promoters. Promoters were generated with increasing spacing between TFBS as represented in (**A)**. Resulting promoters harbouring AP-1 sites were examined in transfected 293T cells that were unstimulated (black bars), stimulated with PMA (red bars) in (**B)** and with fold change illustrated in (**C)**. The same spacing arrangements were assessed with NF-κB sites in transfected cells stimulated with TNFα (green bars) (**D)** with fold changes shown in (**E)**. Promoters with the same spacing and containing the same number of NF-κB sites (8 in total) were also assessed in TNFα stimulated 293T cells to eliminate any influence of binding site number on luciferase activity results (**F)** and fold induction shown **(G)**. Data in F was analysed by 2 way ANOVA to take into account the effect of the change in spacing and the effect of stimulation upon the promoters. This analysis revealed that both factors had significant effects on promoter activity. A post-hoc Šidak test was then performed to compare the stimulated and unstimulated activity of each promoter and significant differences of p < 0.05 are indicated by ★.

We also explored the optimal spacing between neighbouring TFBSs on gene expression. Promoters were assembled containing either NF-κB or AP-1 binding sites, separated by 15 bp to 60 bp space (Fig. 4A). Recombinant plasmids containing these responsive promoters were transiently transfected into 293T cells and were either unstimulated or stimulated with PMA or TNFα to activate AP-1 or NF-κB, respectively (Fig. 4B–E). In both promoter libraries, the promoters with the shortest space (15 bp) between TFBS displayed the highest basal and induced luciferase gene expression compared to promoters with TFBSs separated by a greater distance. In these initial experiments, the number of TFBSs was not standardised, which could potentially influence the outcome of the experiment. We therefore compared the activity of synthetic promoters comprising the same number of TFBSs (8xNF-κB motifs) within each spacer group. Transcriptional activity with the 8xNF-κB-synthetic promoters also complied with the observed trend of higher basal activity and higher induced expression when TFBS were closer together compared to further apart (Fig. 4F and G). As 20 bp spacing between TFBSs was the shortest distance that allowed low basal activity and high induced luciferase expression, we applied this spacing to subsequent promoters.

### Function of optimally spaced TFBS clusters and the effect of multi-stimulation

**Figure 5:**
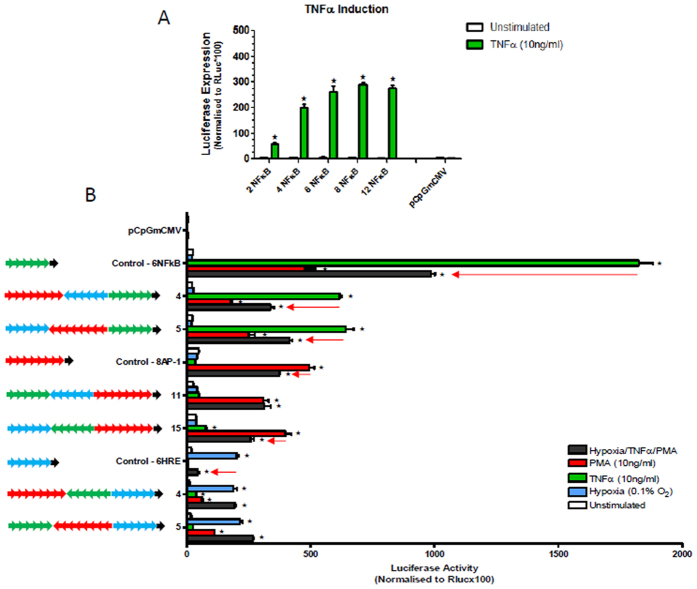
Optimised TFBS number and responsiveness of cluster promoters. The influence of increasing the number of NF-κB sites on promoter responsiveness to TNFα was assessed in transfected 293T cells as illustrated in A (white bars unstimulated, green bars TNFα stimulated). Cluster composite promoters were generated with each type of cluster (NF-κB, AP-1 and HRE) in the proximal position. Basal activity and stimulated activity of these promoters is shown in (**B)**. Data in (**A)** was analysed by 2 way ANOVA to take into account the effect of the change in number of NFkB clusters and the effect of stimulation upon the promoters. This analysis revealed that both factors had significant effects on promoter activity. A post-hoc Šidak test was then performed to compare the stimulated and non-stimulated activity of each promoter and significant differences of p < 0.05 are indicated by ★. The responsiveness of each individual cluster promoter in (**B)** to stimulation was analysed by 1 way ANOVA. A post-hoc Šidak test was then performed to determine the level of significance of mean values above the unstimulated promoter activity. Where the difference from baseline promoter activity was significantly (p < 0.05) different this is indicated by ★. The post-hoc Šidak test also revealed when combined stimulation resulted in a significantly (p < 0.05) lower promoter activation than achieved with the optimal individual stimulation and is illustrated by red arrows.

Endogenous promoters often contain repeats or clusters of the same TFBSs in close arrangements and this feature is thought to promote recruitment of the cognate TF and thus increase the transcription activity of the promoter. We therefore examined the influence of the number of NF-κB binding sites on luciferase gene expression, following TNFα stimulation. The data in Fig. 5A clearly shows that with increasing number of TFBSs, there is a significant increase in TNFα-induction and as the number of NF-κB motifs increases beyond 6, the induction levels remained relatively unchanged. Similar cluster promoters were generated with HRE (6xHRE) and AP-1 (8xAP-1) motifs and interestingly, the activity of these mono-responsive cluster promoters was significantly lower upon multiple stimulation compared to their optimal activation with a single stimulus (Supplementary Figure 2), thus supporting the activation profile of the mono-responsive promoters shown in Fig. 1.

### Composite cluster promoters with optimally sized clusters

We then generated composite promoters with clusters of 6xHRE, 6xNF-κB and 8xAP-1. In these promoters, the proximal TFBS cluster was predetermined by the choice of vector for the random cloning with the other two TFBS cluster types. Promoters were sequenced and shown to contain a single repeat of each cluster, with the central cluster orientated in the reverse direction and a total promoter length of 665 bp. Within these promoters, optimal spacing was retained between TFBS but a 46 bp space was introduced between clusters and these promoters displayed basal expression comparable to the parent vector in transfected 293T cells (Fig. 5B). Following cell stimulation, it was apparent that cluster promoters were predominantly activated through the proximal TFBS cluster and displayed less activation via other distal clusters (Fig. 5B). Importantly, combined stimulation did not result in additive induction of these promoters and indeed, several promoters displayed significantly lower induction with combined stimulation (Fig. 5B). These experiments show that the use of pre-constructed clusters did not generate optimally functioning multi-responsive promoters. These experiments also demonstrate that TNFα and hypoxia were specific for NF-κB and HRE induction respectively, whereas PMA gave efficient activation of AP-1 and to a lesser extent NF-κB. This activation of NF-κB by PMA is in agreement with published literature on PMA^31^,^32^

### Randomly generated composite synthetic promoters with the optimal spatial arrangement of TFBSs exhibit low basal activity, multi responsiveness and additive induction

**Figure 6:**
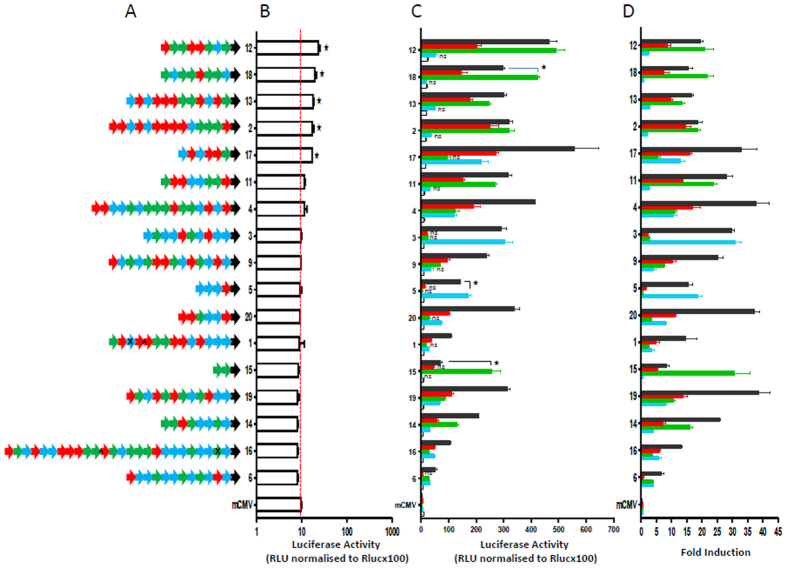
Organisation and function of composite promoters with randomly arranged TFBS. Generated promoters were sequenced and the arrangement of TFBS is schematically illustrated in (**A)** (TFBS: AP-1 (red), NF-κB (green), HRE (blue) the arrow direction indicates the orientation of TFBS and the black arrow represents the mCMV promoter; **X** indicates TFBS error and **^** indicates site of spacing error). The basal activity of the promoters in unstimulated transfected 293T cells is shown in (**B)**. Promoters were induced by stimulation of transfected cells with hypoxia (0.1%), TNFα, PMA and the three combined. Levels of luciferase activity are shown in (**C)** and fold changes in expression illustrated in D. Comparisons of basal expression of all promoters with the mCMV group in (**B)** was performed by 1 way ANOVA which showed significant differences. A post-hoc Dunnett test was then performed to determine significant differences from the basal activity of the mCMV promoter and where p < 0.05 this is indicated by ★. The responsiveness of each individual promoter in (**C)** to stimulation was also analysed by 1 way ANOVA. A post-hoc Šidak test was then performed to determine the level of significance of mean values above the unstimulated promoter. Where the difference from unstimulated promoter activity was not significant (p > 0.05) this is indicated by ns. When the activity with combined stimulation (grey bars was significantly (p < 0.05) lower than the highest activation of the same promoter by an individual stimuli this is indicated by ★.

We then constructed composite synthetic promoters with optimal 20 bp spacing between randomly arranged NF-κB, AP-1 and HIF-1α motifs and a 66 bp space between the proximal TFBS and the TATA box. The overall incorporation rate of the three TFBSs in the 17 promoters sequenced was 35% HRE, 33% AP-1 and 32% NF-κB, which suggests there was no bias in TFBS inclusion. The median number of TFBSs included in the promoters was 10.6 (range 2–26) which is an approximate length of 384 bp and promoters were generally error free with only 2 TFBSs and 2 spacing distances altered (Fig. 6A; Supplementary Material Appendix). Basal expression from all 20bp-composite synthetic promoters was low with an average of 12.1 RLU, which was only significantly higher than the mCMV alone (9.5 RLU) in five promoters (Fig. 6B). Upon stimulation with hypoxia, TNFα or PMA, the promoters displayed appropriate induction based upon their sequence composition (Fig. 6C,D) and only 3 promoters (5, 15 and 18) displayed significantly lower induction with combined stimulation. Sequence analysis of the promoters supported the activation profiles of the promoters, for example the partial or non-responsiveness of promoters - 5, 14 and 18 may have been due to the presence of a single AP-1 site thus resulting in low PMA induction, whereas promoter 5 was devoid of NF-κB sites and was non-responsive to TNFα. Importantly, when all three stimuli were combined, the majority of the composite promoters (1, 4, 6, 9, 14, 16, 17, 19 and 20) displayed additive or synergistic induction.

### Characterisation of dose- and time-dependent induction of luciferase gene expression following genomic integration of composite synthetic promoters

**Figure 7:**
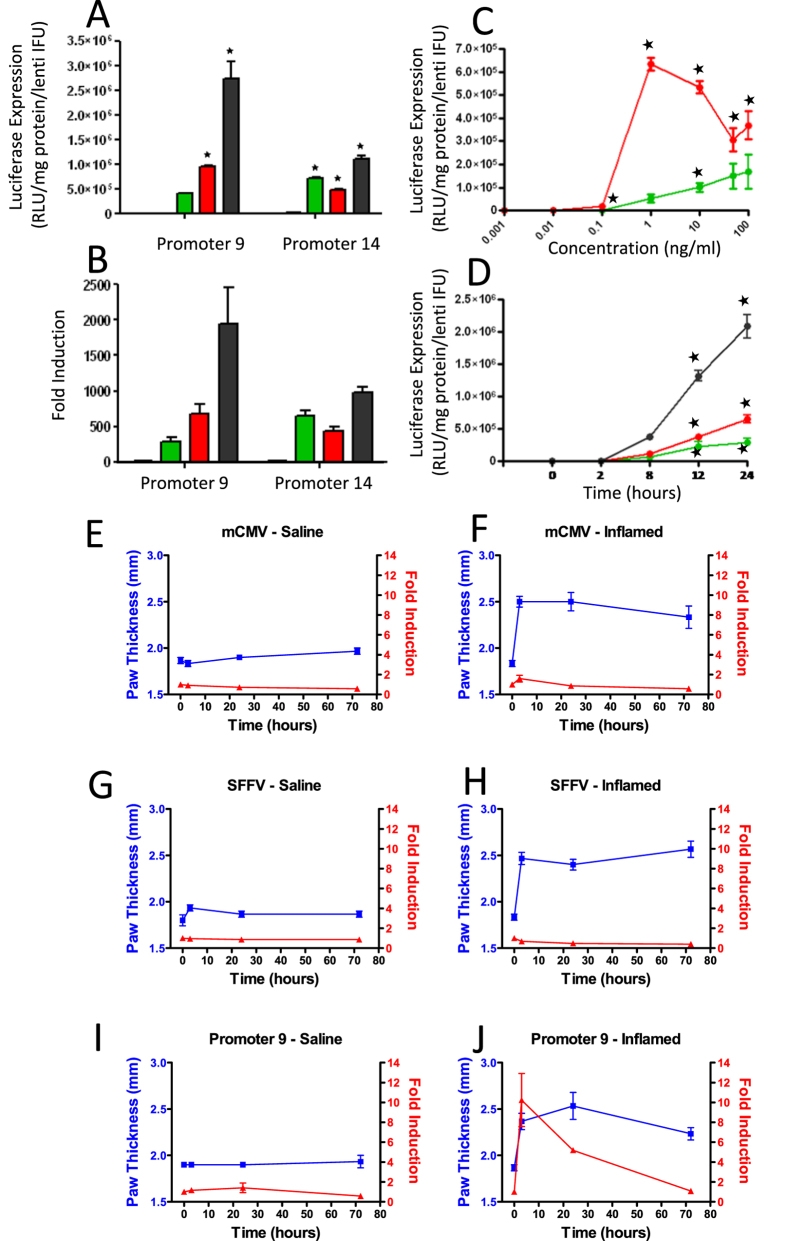
Promoter activity following lentiviral delivery *in vitro* and *in vivo*. 293T cells lentivirally transduced to habour promoters 9 and 14 were responsive to stimulation (A – Green/TNFα; Red/PMA and Black their combination) with fold induction shown in (**B)**. Transduced 293T cells with promoter 9 were used to study dose responsiveness of the promoter to increasing concentrations of TNFα (Green line) and (Red line) PMA (**C)**. The kinetics of activation of promoter 9 were explored at time points up to 24 hours after promoter stimulation with TNFα, PMA and the combination of the two (black line) (**D)**. Activity of promoter 9 was then compared to the mCMV and constitutive SFFV promoter in a mouse paw inflammation model. Expression of luciferase was monitored in whole mice by non-invasive bioluminescent imaging a week after delivery of the virus (time 0) and at intervals after induction of inflammation in the left hind paw by injection of λ–carrageenan. Bioluminescence imaging results are shown in Supplementary Figure 4 and both fold change in bioluminescence from the time 0 baseline (red line) and paw thickness (blue line) were plotted for control (**E,G,I**) and inflamed paws (**F,H,J**). Multiple comparisons of mean data in (**A,C,D**) was analysed by 1 way ANOVA which showed significant differences. A post-hoc Dunnett test was then performed to determine significant increases above unstimulated promoter activity with p values less than 0.05 indicated by ★. Data presented in E to I was analysed for each mouse using Graphpad Prism. Area under the curve was calculated for paw thickness and bioluminescence and then a 2-ANOVA was performed which confirmed there were significant effects of time and treatment. A post-hoc Bonferroni test confirmed a significant (p < 0.05) decline in the bioluminescence from the SFFV promoter in the inflamed paw compared to the saline control and that activity of promoter 9 was significantly (p < 0.05) increased in the inflamed paw compared to the saline control.

Using a lentiviral vector, we examined the responsiveness of promoters 9 and 14 following genomic integration in 293T cells. The promoter sequences and luciferase gene were cloned into a self-inactivating lentiviral vector containing the scaffold attachment region HS4 which insulates the expression cassette from external influences. The promoters retained the same activation profile following genomic integration but with a dramatically increased fold induction (Fig. 7A,B). The activity of promoter 9 was further explored in kinetic studies which revealed interesting differences between PMA and TNFα stimulation; promoter activation with PMA was observed as ‘all-or-nothing’ with significant and maximal activation with 1 ng/ml PMA (1390.8 fold) (Fig. 7C). In contrast, dose-dependent responsiveness was observed with TNFα induction from 100 pg/ml, with a peak induction observed between 50–100 ng/ml TNFα (146.6 fold) (Fig. 7C). Despite these differences, the kinetics of activation with 10 ng/ml of TNFα or PMA were similar, with promoter 9 activated after 8 hours of stimulation and reached peak induction between 24 and 48 hours (Fig. 7D). Importantly, the promoter displayed robust synergistic activation at almost all time-points with combined TNFα and PMA stimulation (Fig. 7D). Lentivirus transduced cells were maintained in culture for 40 days and retained stable levels of promoter function over this time frame (Supplementary Figure 3).

### Composite synthetic promoters display disease-specific induction of luciferase gene expression *in vivo*

The *in vivo* disease-activation profile of promoter 9 delivered with a lentiviral vector was assessed in a carrageenan-induced paw inflammation model. Equal titres of lentiviruses encoding the luciferase gene driven by composite promoter 9, the constitutive promoter SFFV (positive control), or the mCMV (negative control) were injected intraplantarly into both hind paws of mice (n = 3) seven days before carrageenan administration to allow for genomic integration. Paw inflammation was induced by an intraplantar injection of 1% λ-carrageenan suspension into the left hind paw whilst an equal volume of sterile saline was injected into the control right hind paw. Luciferase gene expression was measured using real-time bioluminescence imaging (Supplementary Figure 4) before inflammation (0 hours) and at 3, 24 and 72 hours post-carrageenan injection (Fig. 7E–J) and paw thickness was measured up to 96 hours (Fig. 7E–J). As anticipated, at 0 hours (before inflammation) basal luciferase expression from composite promoter 9 was low and not significantly different from expression levels from the negative control promoter LV-mCMV (Supplementary Figure 5). Following carrageenan injection in the left hind paw, promoter 9 induced robust inflammation-specific luciferase expression which was significantly higher than in the control saline paw when assessed as area under the curve (Fig. 7I,J) The induction of promoter 9 peaked after 3 hours at a level comparable with the SFFV promoter, whilst-paw thickness increased to a peak after 24–48 hours post-carrageenan injection (Fig. 7FH,J). By 72 hours, inflammation was generally lower than at the previous time points but had not returned to baseline and the luciferase expression from the composite promoter had also declined. A contributory factor in the decline of luciferase activity was that increased paw thickness which could reduce photon emission from the luciferase enzyme, as observed in the SFFV promoter group (Supplementary Figure 6).

## Discussion

Regulated synthetic promoters have potential applications in gene therapy of diseases where changes in gene expression can be attributed to defined TF activation. In order for these promoters to function optimally they will need to be responsive to all phases of disease activity and remain active whilst evidence of disease pathology persists. Previously described promoters containing a single TFBS type are mono-responsive^19^,^20^,^21^ so are unlikely to display an activation profile that mirrors disease course. Furthermore, observations in our study show that the activity of such mono-responsive promoters is compromised when multiple TFs are activated during combined stimulation. Importantly, this also included mono-responsive promoters constructed with the optimal spatial arrangement defined in this study. These observations may also provide an important caveat to the more general use of responsive promoters in the monitoring of TF activity. Ideally, disease-responsive promoters should be responsive to any TF involved in the disease pathology and should be maximally induced by each individual TF or all TFs, where the latter results in additive gene expression. By integrating molecular biology and computational bioinformatic studies of TFs, it should be feasible to delineate and exploit all transcriptional pathways activated in a particular condition for gene therapy application.

Core promoter motifs such as TATA boxes are integral to eukaryotic transcription as they serve as platforms for the assembly of the transcriptional preinitiation complex (PIC), comprised of RNA pol II and its basal TFs. The formation of this PIC is sufficient for a basal level of transcription, however, regulated transcription is dependent on the recruitment of gene-, stimuli- or tissue-specific TFs to their cognate binding sites in the promoter regions of target genes^33^. In line with our observations, many studies have also demonstrated that expanding the distance between upstream elements i.e. TFBSs and the TATA box can reduce transcriptional activation^34^,^35^,^36^,^37^,^38^,^39^,^40^,^41^. Taken together, these publications suggest that the transcription initiation process requires specific alignments between TFBSs and the proteins assembled on the TATA box and it is likely that positioning the proximal TFBS closer to the TATA box facilitates their interactions, which favour high gene expression. In designing regulated promoters our 66 bp spacing from the TATA box proved optimal for maintaining low basal activity but retention of high inducibility.

Surprisingly little exploration of the influence of TFBS spatial arrangements on promoter activity have been performed. Most previous work has been on spacing between TFBS in constitutive synthetic promoters with evidence that closely aligned TFBS can result in steric hindrance. Observations in yeast with synthetic GAL4 promoters have shown reduced expression when 2 adjacent sites were separated by 1 bp and a weaker effect was observed for Gcn4 sites positioned 5 bp apart^40^. Increasing the spacing between TFBS has also been shown to influence the activity of synthetic constitutive promoters, activity of a Gal4-VP16 promoter declined when spacing between two binding sites was increased from 0 to 48 bp^42^. Similarly, our studies revealed that spacing between TFBS also influenced promoter induction. Firstly, basal activity was elevated with closer (4 bp and 15 bp) spacing between TFBS but was lower when the spacing was 20 bp or more, regardless of the TFBS arrangement monitored. These observations suggest that the architecture of the DNA sequence with closely aligned TFBS has an innate ability to drive gene expression in the absence of cell stimulation. In the unstimulated state levels of activated TFs will be low and an observation made in *E.Coli* that may be relevant, is that clusters of TFBS (two or more sites) are able to enhance TF binding rates at low concentrations^43^. With closer spacing (15 bp) in PCR assembled promoters there were also increased levels of induced expression and this also declined as spacing was increased (up to 45 bp). Because we were interested in designing promoters with a low basal expression and high induction we opted for spacing of 20 bp between TFBS in subsequent composite promoters. This spacing equates to a distance of 26 to 30 bp between the centre of adjacent TFBS which is between 2 and 3 turns of the DNA helix (20.9 and 31.35 bp respectively) and will position adjacent TFBS on different planar surfaces of the DNA helix.

Many highly active, endogenous human promoters possess clusters of the same type of TFBS^44^ and studies on the theory of facilitated diffusion of TFs indicate that ‘homotypic’ clusters facilitate the recruitment of TFs during their one-dimensional random search on the DNA for cognate TFBSs^45^,^46^,^47^,^48^,^49^. We therefore anticipated that promoters generated with clusters of the same TFBS would exhibit high induction. We observed that increasing the number of clustered TFBSs resulted in a gene expression plateau, suggesting that synthetic promoters can become saturated with TFs, as also demonstrated in hypoxia-responsive synthetic promoters^21^. However, the function of the composite clustered promoters was not optimal because they were predominantly activated through the proximal TFBS cluster and thus displayed lower induction when other distal TFBS clusters in the promoter were activated. Furthermore, following combined stimulation, the composite clustered promoters failed to display additive induction, therefore additional studies are required to determine the optimal arrangements of clustered TFBSs within synthetic promoters to achieve the desired expression profile.

The composite promoters created using Assembly PCR generally displayed low basal activity and were appropriately responsive to individual stimuli. In many cases, they displayed additive induction or synergistic activation following combined stimulation (Fig. 6). Although the number of promoters characterised *in vitro* is small, it is apparent that the best functioning promoters had an even proportion of HRE, AP-1 and NF-κB motifs distributed randomly throughout the promoter rather than containing clusters of homotypic TFBS. With high throughput analysis it should be possible to establish mathematical models for promoter behaviour which will permit the design of promoters with desired functionality. In addition, such modelling may permit better interpretation of endogenous promoter activation and better understanding of mechanisms involved in gene regulation.

More detailed analysis of synthetic promoter function was undertaken following integration into the genome by lentiviral transduction. The dose responsiveness of composite promoter 9 to TNFα stimulation was similar to observations made with homotypic NF-κB promoters^50^. Interestingly, the magnitude of induction was dramatically improved when integrated into the genome with a lentiviral vector. There are several potential causal factors: a) the CpG motifs in the plasmid DNA can activate cells and thus promote higher basal activity and so reduce the magnitude of promoter activation^51^ b) the innate preference of lentiviruses to integrate into transcriptionally active sites^52^ and c) the use of HS4 insulation elements which bound the integrated promoter, has previously been shown to increase the activity of lentivirus integrated promoters due to better transcriptional termination^53^,^54^. In addition, the HS4 elements may also contribute to maintaining promoter function, as the HS4 insulators and bounded transgenes become acetylated and this modification is thought to be important in preventing methylation over the promoter^55^. Lentivirally integrated promoter 9 was shown to be highly sensitive, rapidly activated and exhibited dose- and time-dependent responses to stimulation.

*In vivo* studies with local delivery of promoter 9 in mouse paws confirmed disease-specific induction of luciferase gene expression following carrageenan-induced paw inflammation, as determined by real-time bioluminescence imaging. These observations confirm that locally activated TFs at the disease site are able to activate the promoter in a disease specific manner. Activity of the promoter peaked at 3 hours and was lower when inflammation peaked at 24 to 48 hours, however, observations with the SFFV promoter group suggest that the increased tissue thickness in the inflamed paws negatively impacts on the luciferase light emission which would lead to an underestimation of the promoter activity in inflamed tissue which we have previously suggested^23^. Further studies are therefore required to determine how closely promoter activation mirrors the profile of paw inflammation.

The use of assembly PCR to generate composite synthetic promoters has several advantages as it permits randomness in TFBS arrangement and primers can be designed to include diversity within the consensus sequence of TFBS, in addition the length of assembled promoters can be broadly controlled through the concentration of stop oligonucleotides used in assembly reactions. Several generated promoters with optimised spatial arrangement had the functional characteristics we desired – compact, low basal activity, multi-responsiveness and additive induction. The assembly process and spacing arrangements described are also applicable to synthetic biology where promoters are used as logic gates. We have generated promoters with OR logic (promoter 4) which was similarly responsive to all three stimuli whilst other promoters were closer to AND logic (promoter 1). Clearly the spacing and screening was designed to select for promoter activation but could be optimised for the selection of other logic gates.

## Methods

### Cell culture, transient transfection and cell stimulation

Human embryonic kidney 293T (HEK293T) cells were grown in Dulbecco’s Modified Eagle’s Medium (DMEM) (Lonza Group Ltd., Switzerland) supplemented with 10% heat-inactivated foetal bovine serum (FBS; Gibco, Life Technologies Corp, California, USA), 4.5g/L glucose (Sigma Aldrich, Corp, St Louis, MO, USA), 100 IU/ml penicillin (Sigma Aldrich), 100 μg/ml streptomycin (Sigma Aldrich) and 2 mM L-glutamine (Sigma Aldrich) and were maintained at 37 °C in a humidified 10% CO_2_ incubator. Twenty-four hours prior to transfection, 293T cells were seeded at a density of 2 × 10^4^ cells in 96-well plates containing DMEM supplemented with 10% FBS and antibiotics. After 24 hours, the cells were co-transfected in triplicate with a total concentration of 200 ng/well DNA (180 ng firefly luciferase expressing recombinant plasmid DNA and 20 ng renilla luciferase expressing plasmid (pRL-CMV; Promega Corp., Madison, WI)) using FuGENE 6 Transfection Reagent (Promega Corp). Following 24 hours post-transfection, the cell medium was replaced with DMEM containing 0.5% FBS alone for the unstimulated control cells and incubated in normal conditions for 18 hours. For the activation of HIF-1α, DMEM containing 0.5% FBS alone was added to the cells and the plate was incubated in hypoxic conditions at 0.1% O_2_, 10% CO_2_ and 89.9% N_2_ in a CO_2_ incubator (New Brunswick, Hamburg, Germany) for 18 hours. For the activation of inflammation-responsive TFs and/or all TFs, the cells in DMEM containing 0.5% FBS were stimulated with 10 ng/ml human TNFα (Activation of NF-kB -Peprotech, NJ, USA) or 10 ng/ml PMA (Activation of AP-1 - Sigma Aldrich) or a combination of 10 ng/ml TNFα and 10 ng/ml PMA in 0.1% hypoxia for 18 hours.

### Assembly PCR method

A detailed description of the Assembly PCR method, including the subsequent construction of synthetic promoters in plasmids, lentiviral vectors and the generation of lentiviruses is described in the supplementary material.

### Lentiviral stable cell lines and cell stimulation

Human kidney 293T cells (5 × 10^4^) were seeded in complete DMEM medium in a 6-well plate, 24 hours before lentivirus transduction. Lentivirus particles (approximately 3.5 × 10^5^ IFU) were added to the cells supplemented with 6 μg/ml polybrene and incubated for 72 hours, after which the transduced cells were trypsinised and cultured in complete DMEM using standard protocols. For the dose-response experiments, the transduced 293T cells (2 × 10^4^ in 96-well plates) either remained unstimulated or were stimulated with 0.001, 0.01, 0.1, 1, 10, 50 or 100 ng/ml of PMA or TNFα for 18 hours. For the time course experiments, transduced cells (2 ×10^4^ in 96-well plates) were either unstimulated or stimulated with 10 ng/ml PMA, 10 ng/ml TNFα or their combination for 2, 8, 12 and 24 hours. Firefly luciferase expression was normalised to the total protein content in the same sample and also to the titre of the respective lentivirus and the data was expressed as RLU/mg protein/lenti IFU.

### Luciferase assay and BCA assay

The luciferase activity in transiently transfected and transduced cell lysates was measured 18 hours post-stimulation by removing cell medium and lysing the cells in 50 μl of 1× Passive Lysis Buffer (Promega) or 50 μl of 1× Glo Lysis Buffer (Promega), respectively, at room-temperature for 15 minutes with orbital shaking. The intracellular firefly and renilla luciferase expression in the same samples were quantified using the Luciferase Assay System (Promega) and the Renilla Luciferase Assay System (Promega), respectively, following the manufacturer’s instructions with minor modifications. The luciferase data was expressed as relative light units (RLU), as determined by the MLX Microtiter Plate Luminometer and Revelation software (Dynex Technologies Inc, Chantilly, VA, USA). The total protein concentration in transduced cell lysates was quantified using the Pierce BCA Protein Assay Kit (Thermo Scientific, Rockford, IL, USA) as per the manufacturer’s protocol and absorbance measurements at 562 nm were performed using a Tecan Genios Microplate Reader (Tecan Group Ltd, Männedorf, Switzerland) and Magellan 4 software. Firefly luciferase values from transduced cells were normalised to the total protein concentration in the same samples and expressed as relative light units per milligram of protein (RLU/mg).

### Intraplantar delivery of lentiviruses to mouse paws

All experiments with adult male CD1 mice (6–8 weeks old) were approved and performed under Home Office regulations (Scientific Procedures Act 1986) and *in vivo* lentivirus experiments were performed under strict biological containment. Mice were anaesthetised with AErrane (Isofluorane; Baxter Healthcare Ltd, Thetford, Norfolk) using Boyle’s apparatus (British Oxygen Company BOC, London, UK) and 25 μl of the lentiviruses, LV.9.Luc^+^, LV.mCMV.Luc^+^ or LV.SFFV.Luc^+^, containing 260,000 lentivirus IFU in DMEM, were injected intraplantarly into both hind paws of the mouse and allowed to integrate into the cell genome for 7 days.

### Carrageenan-induced paw inflammation model

Seven days post-lentivirus injection, inflammation was induced in the left hind paws of mice by intraplantar injection with 50 μl of 1% λ-carrageenan (Sigma Aldrich) in saline (w/v) and the right hind paws received an equivalent volume of sterile saline, as control. Paw thickness, was measured at 0, 3, 24, 48 and 72 hours post-carrageenan injection using POCO 2T calipers (Kroeplin Längenmesstechnik, Schlüchtern, Germany).

### *In vivo* bioluminescence imaging

Whole body luciferase activity in mice was non-invasively monitored at 0, 3, 24 and 72 hours post-carrageenan injection using real-time bioluminescence imaging. Mice received an intraperitoneal injection with 200 μl of luciferin K^+^ Salt (30 mg/ml; Promega Corp) and were anaesthetised with isofluorane 15 minutes before imaging. Anaesthetised mice were photographed (0.2-second exposure) and imaged for light emission (5 minutes on medium sensitivity) with the IVIS 1000 series (Caliper Life Sciences Corp, Hopkinton, MA, USA). Bioluminescence images were overlaid on gray-scale photographs which were obtained with a 12-cm field of view, a binning of 8 and a 1/*f* stop and open filter. The regions of interest (ROI) were defined manually over both hind paws and the background photon flux was defined in control regions of the same size, to obtain quantitative photon data. Light emission was quantified as photons per steradian per square centimetre (photons/second/steradian/cm^2^/) using Living Image Software (Caliper Life Sciences Corp).

### Statistical Analysis

Statistical analysis was performed using SPSS software (IBM SPSS Statistics 23), Graphpad Prism 4 version 4.03 (GraphPad Software, Inc., La Jolla, CA, USA) and Microsoft Excel (Microsoft Corp, Redmond, WA, USA). The statistical tests performed on each data set are described in the relevant figure legends.

## Additional Information

**How to cite this article**: Mohamed, H. *et al*. Assembly PCR synthesis of optimally designed, compact, multi-responsive promoters suited to gene therapy application. *Sci. Rep.* **6**, 29388; doi: 10.1038/srep29388 (2016).

## Supplementary Material

Supplementary Information
