# Supplementary material for: Assembly PCR synthesis of optimally designed, compact, multi-responsive promoters suited to gene therapy application
Source: Sci Rep. 2016 Jul 8;6:29388. doi: 10.1038/srep29388 (PMC4937410; doi:10.1038/srep29388)
Supplement: Supplementary Information [file srep29388-s1.pdf]

Supplementary Material

Assembly PCR synthesis of optimally designed, compact, multi-responsive promoters suited to gene therapy application.

Mohamed H, Chernajovsky Y and Gould D

Supplementary Methods

Assembly PCR reactions were performed using the G-Storm Thermocycler (model GS482, Gene Technologies Limited, Essex, UK) and all oligonucleotides were manufactured by Sigma-Aldrich with standard desalting purification (listed in Supplementary Table 2). Each forward and reverse TFBS-oligonucleotide contained the core binding sites of NF-κB, HIF-1α or AP-1 flanked by annealing sequences of 10 bp, 15 bp or 20 bp. Spacer reverse oligonucleotides contained 5 nt or 10 nt spacer sequences flanked by annealing sequences of 15 bp or 20 bp. The 5'-Stop-NheI primers and 3'-Stop-XhoI contained NheI and XhoI restriction enzyme sites, respectively and annealing sequences of 10 bp, 15 bp or 20 bp.

For the initial assembly (x10 cycle) PCR reaction, an oligonucleotide mix containing 5 µl 100 µM forward TFBS-oligonucleotide, 5 µl 100 µM reverse TFBS-oligonucleotide, 12.8 µl 100 µM 5'-Stop-NheI and 12.8 µl 100 µM 3'-Stop-XhoI oligonucleotides in a final reaction volume of 35.6 µl, was diluted 1:100 in sterile distilled (d)H_2O. A volume equal to the final reaction volume before dilution was added to the assembly PCR reaction e.g. 35.6 µl diluted oligonucleotide mix was added to 12 µl 5x Phusion HF Buffer (NEB), 6 µl 2 mM dNTPs, 0.5 µl Phusion DNA Polymerase (NEB) and sterile dH_2O in a final volume of 60 µl. The oligonucleotides were assembled into double stranded PCR products in a ten cycle PCR reaction using the thermocycler program: 95°C for 5 minutes, 10 cycles of 95°C for 30 seconds, 60°C for 45 seconds, 72°C for 45 seconds and an additional extension at 72°C for 2 minutes. The reaction was purified from excess dNTPs and free oligonucleotides using the PureLink® PCR Purification Kit (Invitrogen Corp., Paisley, UK) to yield the purified double stranded assembled PCR product which served as the template for the amplification PCR reaction (x25 cycles). The amplification reaction comprised 15 µl of assembled PCR product, 10 µl 5x Phusion HF Buffer, 0.5 µl Phusion DNA Polymerase, 5 µl 2 mM dNTPs, 5 µl 10 µM 5'-Stop-Nhe I, 5 µl 10µM 3'-Stop-Xho I oligonucleotides and sterile dH_2O to a final volume of 50 µl. The PCR products were amplified using the thermocycler program: 95°C for 5 minutes, 25 cycles of 95°C for 30 seconds, 60°C for 45 seconds, 72°C for 45 seconds and an additional extension at 72°C for 5 minutes and then purified by two successive high cut-off PCR purification steps using the Binding Buffer HC provided in the PureLink® PCR Purification Kit (Invitrogen) to remove the amplification primers and failed PCR products <300bp. The purified PCR products were digested with Nhe I and Xho I and cloned into the compatible sites within pCpGmCMV (see below).

The PCR products required to generate the constructs with specific spacing between the proximal TFBS and the TATA box were assembled in four individual reactions comprising 5 µl 100 µM forward HRE-oligonucleotide, 5 µl 100 µM reverse HRE-oligonucleotide, 12.8 µl 100 µM 5'-Stop-Nhe I and 12.8 µl 100 µM of either 3'-Stop-XhoI-0 bp-SalI, 3'-Stop-XhoI-5 bp-Sal I, 3'-Stop-XhoI-9 bp-SalI or 3'-Stop-XhoI-14 bp-SalI-oligonucleotides (Supplementary Table 2). All oligonucleotides contained 20 bp annealing sequences and were assembled, amplified and purified as described above. The PCR products were digested with NheI and SalI and cloned into the NheI/XhoI compatible site within the pCpGmCMV vector to generate the pCpGmCMV-Xbp plasmids, where X bp denotes 0 bp, 5 bp, 9 bp or 14 bp space between the proximal TFBS and the TATA box.

Cloning vectors and construction of synthetic promoters

The pGL3smCMV plasmid (33), containing the human small minimal CMV (mCMV) promoter, equivalent to -52 to -14 of the wild type CMV promoter (60) was digested with NheI and AfeI to isolate the mCMV promoter and firefly luciferase reporter gene (Luc^+). The pCpG-mSEAP plasmid (InvivoGen, San Diego, CA, USA) was digested with NheI, SbfI and XbaI to isolate the fragment containing the nuclear matrix attachment regions from the 5'-portion of the human IFN-β gene and β-globin gene. The digested fragments from pCpG-mSEAP and pGL3mCMV were ligated to generate the pCpGmCMV-Luc^+ construct, which was subsequently digested with NheI and XhoI, to form the pCpGmCMV cloning vector.

Annealed 66 bp-spacer oligonucleotides, containing 5'-XbaI and 3'-XhoI overhangs and an internal NheI restriction site (Supplementary Table 1), were cloned into the compatible NheI/XhoI site within the pCpGmCMV cloning vector and the resulting pCpGmCMV-66 bp plasmid was digested with NheI and dephosphorylated with Calf Intestinal Phosphatase, to form the pCpGmCMV-66 bp cloning vector. The forward and reverse oligonucleotides required for the random ligation cloning method contained the core binding sites of NF-κB, HIF-1α or AP-1 with phosphorylated 5'-CTAG protruding end, compatible to the NheI cleaved end. The pCpG-4bp-composite synthetic promoters were constructed by the random ligation of annealed NF-κB, AP-1 and HIF-1α-oligonucleotides, upstream of the mCMV promoter into the pCpGmCMV-66 bp vector to allow 4 bp space between the TFBSs and 66 bp between the proximal TFBS and the TATA box.

The pCpGmCMV-Xbp plasmids were digested with NheI and XhoI to form the pCpGmCMV-Xbp cloning vectors and the PCR products containing 8xAP-1 or 6xNF-κB were cloned into each cloning vector to allow 55 bp, 60 bp, 66 bp, 70 bp and 74 bp space between the proximal TFBS and the TATA box. The synthetic promoters with 15 bp-60 bp spacing between NF-κB or AP-1 motifs were created by assembling specific combinations of oligonucleotides (listed in Supplementary Table 2). The resulting NheI/XhoI digested PCR products into the pCpGmCMV-5 bp cloning vector, to allow 15 bp-60 bp space between the TFBSs and 66 bp between the proximal TFBS and the TATA box. The clustered composite promoters were assembled by selecting a promoter with the appropriate proximal cluster and digesting it with NheI followed by dephosphorylation and purification. Clusters of 6xNF-κB, 8xAP-1 and 6xHRE were then isolated from the appropriate vectors by digestion with NheI/XhoI, these clusters were then gel purified and two were randomly cloned into the appropriate dephosphorylated vector with the third cluster proximal to the core promoter. The pCpG-20 bp-composite synthetic promoters were constructed by cloning the PCR products, containing NheI and XhoI cleaved ends, into the equivalent sites within the pCpGmCMV-5 bp cloning vector, to allow 20 bp space between the TFBSs and 66 bp between the proximal TFBS and the TATA box.

All pCpGmCMV plasmid constructs were transformed into chemically-competent GT115 cells and selectively grown in 25 μg/ml Zeocin whilst pGL3mCMV and pRL-CMV (Promega Corp.) plasmid constructs were propagated in chemically-competent DH5α cells and selectively grown in the presence of 100μg/ml carbenicillin. Plasmid DNA was isolated from positive bacterial transformants using the PureLink® Quick Plasmid Miniprep or HiPure Maxiprep Kits (Invitrogen) and the selected pCpGmCMV-synthetic promoter plasmids were confirmed by DNA sequencing (Genome Centre, QMUL, UK or GATC-Biotech AG, Germany) and/or restriction digestion.

Construction of lentiviral vectors expressing luciferase

The HIV-1 based, 3'LTR self-inactivating (SIN) lentiviral plasmid pLV.CMVenh.gp91.eGFP.cHS4 (Addgene plasmid 30471; Barde *et al.,* 2011) was digested with BamHI and SalI to release the GFP gene and create the LV.CMV cloning vector. The forward and reverse PCR primers; 5'-GATGAGCAGGATCCCATGGAAGACG-3' and 5'-ATGTACGCGTCGACTCTAGAATTAC-3', containing BamHI and SalI restriction enzyme sites (underlined) respectively, were used to amplify the luciferase gene from pCpGmCMV-Luc$^+$. The PCR product was digested with BamHI and SalI and cloned into the corresponding site within the pLV.CMV cloning vector to incorporate the luciferase gene and create the pLV.CMV.Luc$^+$ plasmid. The CMV promoter and the 5'-portion of the luciferase gene were removed from the LV.CMV.Luc$^+$ plasmid by digestion with PmeI and BstBI to create the LV.Luc$^+$ cloning vector. The candidate synthetic promoters and the 5'-portion of the luciferase gene were amplified from their respective pCpG-20 bp-composite promoter constructs using the forward and reverse PCR primers; 5'-GTCGGATTATCGATGCTAGCGTGCC-3' and 5'-ACTCGTAGTTCGAAGTACTCAGCGT-3', containing ClaI and BstBI restriction enzyme sites (underlined), respectively. The mCMV promoter and the 5'-portion of the luciferase gene were amplified from pGL3mCMV using the forward PCR primer; 5'-GTCGGATTATCGATGCGTGCTAGC-3' and the above reverse primer. All PCR products were digested with ClaI and the 5'-end of the DNA was blunted by incubation with Klenow fragment of DNA polymerase and subsequently digested with BstBI. The PCR products were cloned into the PmeI/BstBI within the LV.Luc$^+$ cloning vector to restore the luciferase gene and incorporate the candidate synthetic promoters or the mCMV negative control promoter to create LV.2.Luc$^+$, LV.9.Luc$^+$ and LV.mCMV.Luc$^+$ transfer plasmids, respectively. The pUCL-Luc$^+$ plasmid (61) was digested with EcoRI and incubated with Klenow to generate 5'-blunt ended DNA, which was digested with BstBI to release the constitutive SFFV promoter and the 5'-portion of the luciferase gene. The resulting fragment was cloned into the compatible PmeI/BstBI site within the LV.Luc$^+$ cloning vector to create the LV.SFFV.Luc$^+$ positive control transfer plasmid.

Production of lentiviruses and transduced cell lines.

Human 293T cells (9 x 10^6) were seeded in complete DMEM medium in a 15 cm^2 tissue culture dish and transiently transfected with three plasmids using polyethyleneimine (PEI) the following day: 18µg of transfer plasmid (above), 18µg of packaging plasmid pCMVΔR8.2 (62), 4µg vesicular-stomatitis virus glycoprotein (VSV-G) envelope plasmid pMD.G (63) were added to OptiMEM medium (Invitrogen) in a final volume of 1ml. Polyethyleneimine (PEI; Sigma Aldrich) was diluted by combining 200µl PEI and 800µl of OptiMEM medium and the DNA:PEI complex was formed by incubating the DNA solution with the diluted PEI for 10 minutes at room temperature. A final concentration of 25 µg/ml chloroquine was added to the cells one hour prior to transfection, after which the transfection mix (2 ml) was added in a drop-wise manner to the 293T cells and incubated at 37°C in 5% CO_2 overnight. The cell medium was replaced with complete DMEM medium the following day and after 72 hours post-transfection, the cell medium containing the packaged lentiviruses were collected and filtered through 0.45µM filters to remove cell debris. The lentiviruses were harvested and concentrated by ultracentrifugation in a Beckman XL-90 Ultracentrifuge (Beckman Coulter, CA, USA) at 23,000 rpm, 4°C for 2 hours and the lentivirus pellets were resuspended in DMEM medium and stored at -80°C. The Lenti-X™ p24 Rapid Titer Kit (Clontech Laboratories, CA, USA) was used to quantify the p24 antigen and therefore, the total viral titre, in

the concentrated lentivirus preparations as per the manufacturer's instructions. The titres obtained were in the range 3.3x10^{7} to 3x10^{8} IFU/ml

	Oligonucleotide Sequences (5'-3')
AP-1 Forward Oligo	5'- CTAG**TGAGTCA** -3'
AP-1 Reverse Oligo	5'- CTAG**TGACTCA** -3'
HRE Forward Oligo	5'- CTAG**ACGTGC** -3'
HRE Reverse Oligo	5'- CTAG**GCACGT** -3'
NF-κB Forward Oligo	5'- CTAG**GGGACTTTCC** -3'
NF-κB Reverse Oligo	5'- CTAG**GGAAAGTCCC** -3'

Supplementary Table 1. List of TFBS-oligonucleotides used to construct the pCpG-4bp-composite synthetic promoter constructs. Overhangs are underlined and TFBSs are highlighted in bold.

Fixed NFκB- 30 bp Forward primer	5'-ATCTCTGCGATGAACCTCACCATGT**GGGACTTTCC**ACAAGGTGCCTCTTATGATCTGGAT-3'
Fixed NFκB- 30 bp Reverse primer	5'- GTGAGGTTCATCGCAGAGATCTTGT**GGAAAGTCCC**ACATGATCCAGATCATAAGAGGCAC-3'
Fixed NFκB- 20 bp Forward primer	5'-ATCTCTGCGATGAACCTCAC**GGGACTTTCC**GTGCCTCTTATGATCTGGAT-3'
Fixed NFκB- 20 bp Reverse primer	5'- GTGAGGTTCATCGCAGAGAT**GGAAAGTCCC**ATCCAGATCATAAGAGGCAC-3'
Fixed NFκB- 15 bp Forward primer	5'-TGCGATGAACCTCAC**GGGACTTTCC**GTGCCTCTTATGATC-3'
Fixed NFκB- 15 bp Reverse primer	5'- GTGAGGTTCATCGCA**GGAAAGTCCC**GATCATAAGAGGCAC-3'
Fixed AP-1- 30 bp Forward primer	5'-ATCTCTGCGATGAACCTCACCATGT**TGAGTCA**ACAAGGTGCCTCTTATGATCTGGAT-3'
Fixed AP-1- 30 bp Reverse primer	5'- GTGAGGTTCATCGCAGAGATCTTGT**TGACTCA**ACATGATCCAGATCATAAGAGGCAC-3'
Fixed AP-1- 20 bp Forward primer	5'-ATCTCTGCGATGAACCTCAC**TGAGTCA**GTGCCTCTTATGATCTGGAT-3'
Fixed AP-1- 20 bp Reverse primer	5'- GTGAGGTTCATCGCAGAGAT**TGACTCA**ATCCAGATCATAAGAGGCAC-3'
Fixed AP-1- 15 bp Forward primer	5'-TGCGATGAACCTCAC**TGAGTCA**GTGCCTCTTATGATC-3'
Fixed AP-1- 15 bp Reverse primer	5'- GTGAGGTTCATCGCA**TGACTCA**GATCATAAGAGGCAC-3'
Fixed HRE- 20 bp Forward primer	5'-ATCTCTGCGATGAACCTCAC**ACGTGC**GTGCCTCTTATGATCTGGAT-3'
Fixed HRE- 20 bp Reverse primer	5'-GTGAGGTTCATCGCAGAGAT**GCACGT**ATCCAGATCATAAGAGGCAC-3'
Fixed HRE- 15 bp Forward primer	5'-TGCGATGAACCTCAC**ACGTGC**GTGCCTCTTATGATC-3'

Fixed HRE- 15 bp<br>Reverse primer	5’-GTGAGGTTCATCGCA**GCACGT**GATCATAAGAGGCAC-3’
Fixed HRE- 10 bp<br>Forward primer	5’-TGAACCTCAC**ACGTGG**GTGCCTCTTA-3’
Fixed HRE- 10 bp<br>Reverse primer	5’- GTGAGGTTCA**GCACGT**TAAGAGGCAC-3’
5nt Spacer -20 bp<br>Reverse primer	5’- GTGAGGTTCATCGCAGAGAT**CATGG**ATCCAGATCATAAGAGGCAC-3’
5nt Spacer -15 bp<br>Reverse primer	5’- GTGAGGTTCATCGCA**CATGG**GATCATAAGAGGCAC-3’
10nt Spacer -20 bp<br>Reverse primer	5’- GTGAGGTTCATCGCAGAGAT**ACAGACATGG**ATCCAGATCATAAGAGGCAC-3’
10nt Spacer -15 bp<br>Reverse primer	5’- GTGAGGTTCATCGCA**ACAGACATGG**GATCATAAGAGGCAC-3’
5’- Stop-NheI<br>20 bp primer	5’-CAGTTGCTAGCGTGCCTCTTATGATCTGGAT-3’
5’- Stop-NheI<br>15 bp primer	5’-CAGTTGCTAGCGTGCCTCTTATGATC-3’
5’- Stop-NheI<br>10 bp primer	5’-CAGTTGCTAGCGTGCCTCTTA-3’
3’- Stop-XhoI<br>20 bp primer	5’-GGATTCTCGAGATCCAGATCATAAGAGGCAC-3’
3’- Stop-XhoI<br>15 bp primer	5’-GGATTCTCGAGGATCATAAGAGGCAC-3’
3’- Stop-XhoI<br>10 bp primer	5’-GGATTCTCGAGTAAGAGGCAC-3’
3’- Stop-XhoI- 0bp-SalI<br>20 bp primer	5’-GGATTGTCGACTCGAGATCCAGATCATAAGAGGCAC-3’
3’- Stop-XhoI- 5bp-SalI<br>20 bp primer	5’-GGATTGTCGACCATGGCTCGAGATCCAGATCATAAGAGGCAC-3’
3’- Stop-XhoI- 9bp-SalI<br>20 bp primer	5’-GGATTGTCGACCAGACATGGCTCGAGATCCAGATCATAAGAGGCAC-3’
3’- Stop-XhoI- 14bp-SalI<br>20 bp primer	5’-GGATTGTCGACGGATACAGACATGGCTCGAGATCCAGATCATAAGAGGCAC-3’

Supplementary Table 2. List of oligonucleotides used for the Assembly PCR method. TFBSs are highlighted in bold. The Nhe I, Xho I or Sal I restriction sites within the ‘Stop’ oligonucleotides are underlined.

Supplementary Results

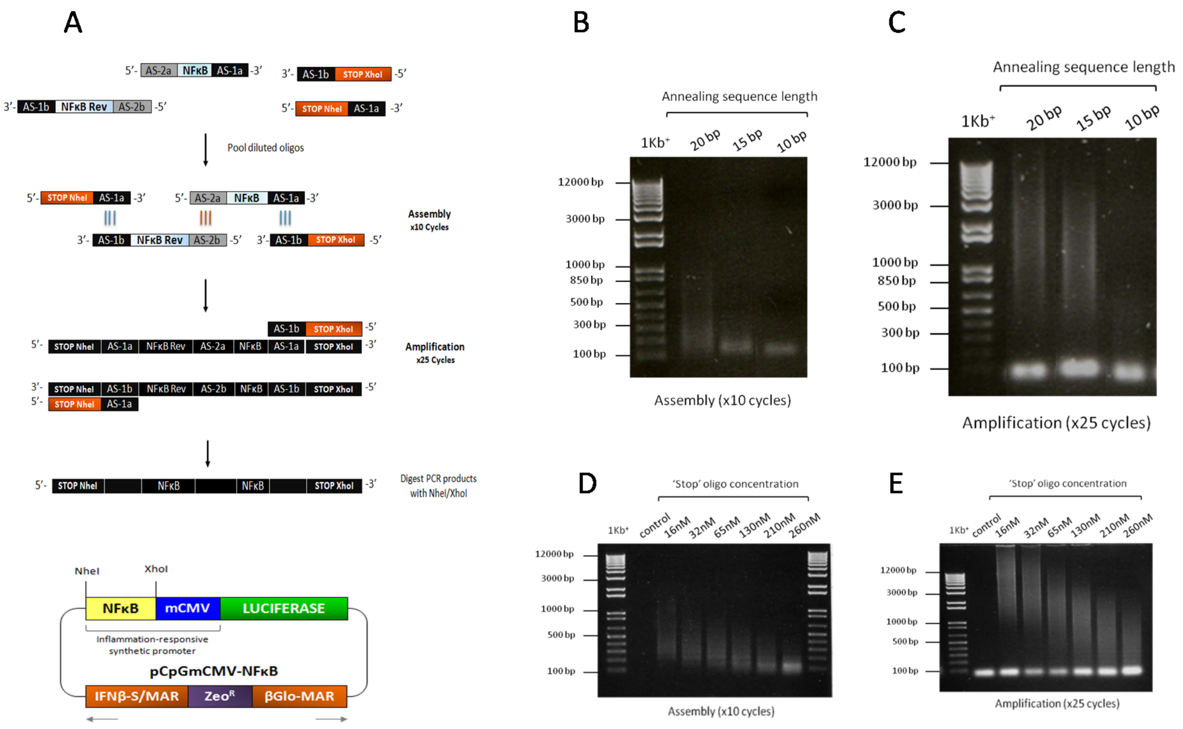

Supplementary Figure 1. Schematic representation of assembly PCR and electrophoresis of obtained products. The stages of synthetic promoter synthesis by assembly PCR include assembly, amplification, and restriction digest with NheI and XhoI prior to cloning into vector pCpGmCMV, these steps are schematically represented in A. The effect of annealing sequence length on the annealing and amplification products are illustrated in B and C respectively and the effect of increasing the 'stop' oligonucleotide concentration in the annealing reaction on assembly and amplification products is shown in D and E respectively .

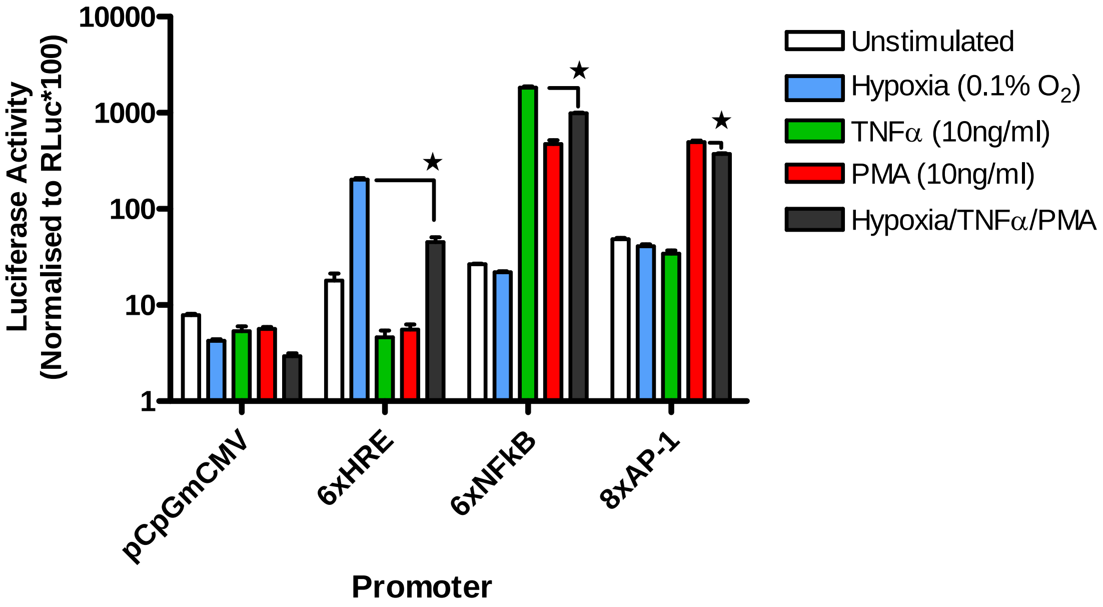

Supplementary Figure 2. Reduced function of optimally arranged singularly responsive promoters following multiple stimulation. The responsiveness of each promoter to stimulation was analysed by 1 way ANOVA (within each promoter) which showed significant differences. A post-hoc Šidak test was then performed to determine when the activity with combined stimulation (grey bars) was significantly ($p<0.05$) lower than the highest activation caused by an individual stimuli this is indicated by ★.

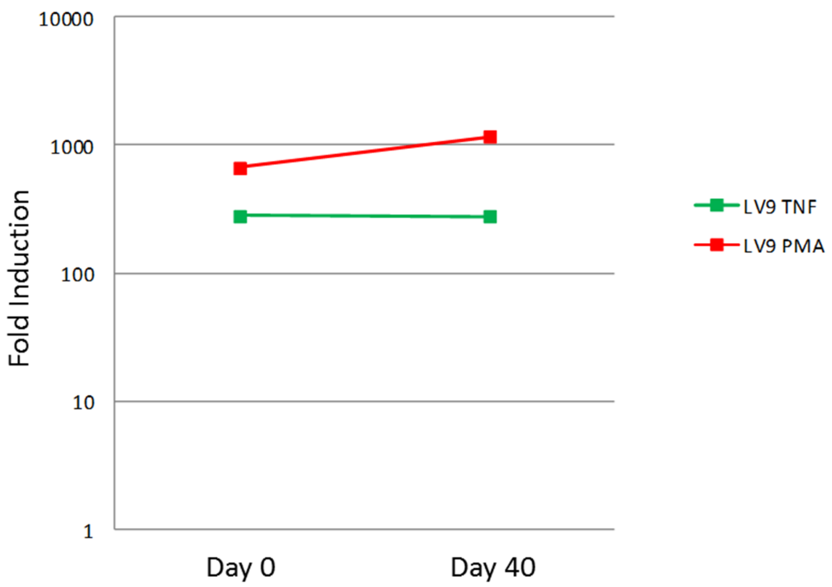

Supplementary Figure 3. Retained promoter activity in transduced cells. Promoter function in lentivirally transduced cells was stable over the 40 day period that cells were maintained in tissue culture.

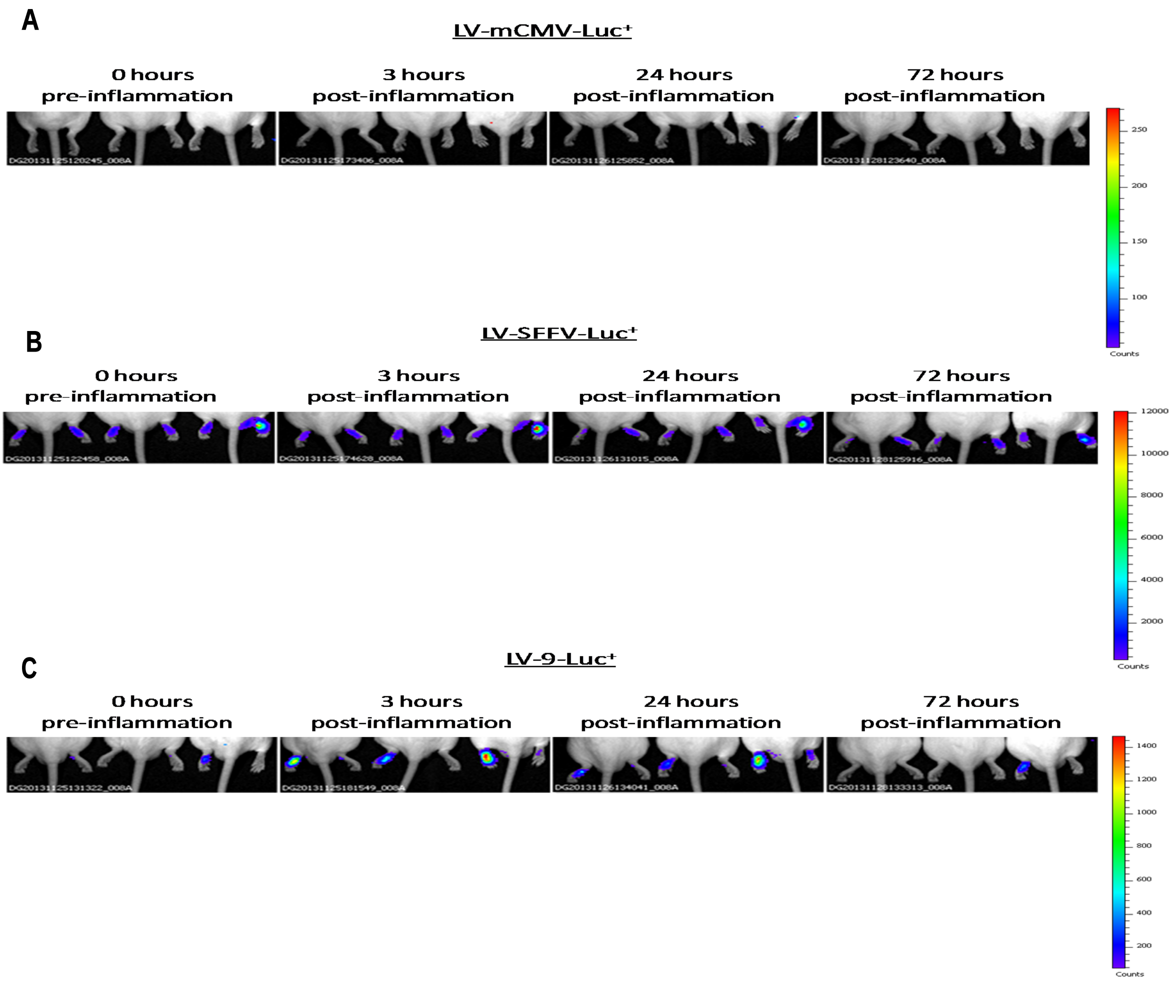

Supplementary Figure 4. *In vivo* bioluminescent imaging of promoter activity. The promoters mCMV (A), SFFV (B) and promoter 9 (C) were delivered to both hind paws in lentiviral vectors in which the drive expression of luciferase. Inflammation was induced in the left hind paw by injection of λ-carrageenan whilst the right hind paw was injected with saline. Groups of 3 animals were used for each promoter and sequential images were collected prior to, and 3, 24 and 72 hours after induction of paw inflammation. Images were collected from above with the anaesthetised mice in a normal posture but with their hind paws exposed.

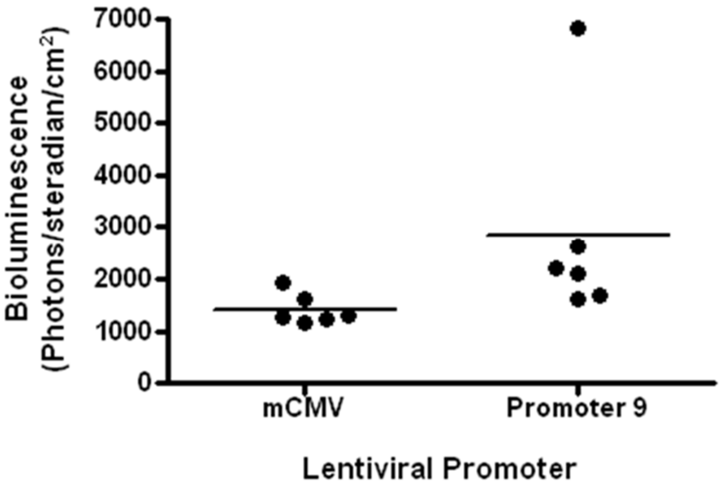

Supplementary Figure 5. Basal promoter activity *in vivo*. Basal promoter activity was determined in all paws by bioluminescent imaging prior to induction of inflammation and was not significantly ($p>0.05$) different when assessed by the student T test.

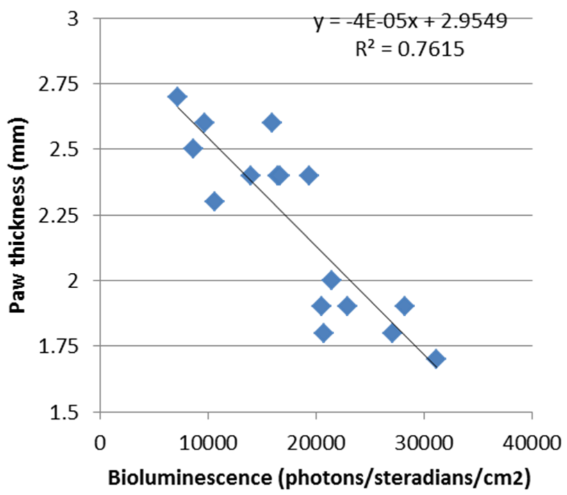

Supplementary Figure 6. Relationship between paw thickness and bioluminescence. Activity of the constitutive SFFV promoter demonstrates that there is a strong negative correlation with paw thickness when assessed at all time points.

Appendix

Composite promoter sequences

Key: **Bold text –** TATAA box

Underlined text – Restriction site

Boxed text – TFBS

Boxed bold text – TFBS error

Double underlined text – spacing error

Compact 4bp Composite Promoters

Promoter 2

AAACCTCCCACACMTCCCCCTGAACCTGAAACATAAAATGAATGCAATTGTTGTTGTTAACTTGTTTATTGCAG
CTTATAATGGTTACAAATAAAGCAATAGCATCACAAATTTCACAAATAAAGCATTTTTTTCACTGCATTCTAGTT
GTGGTTTGTCCAAACTCATCAATGTATCTTATCATGTCTGGCCAGCTAGCCCGGGCTCGAGGCCTGTAGGCGT
GTACGGTG GGAGGCTTATATAAGCAGAGCTCAAGCTGGCATCCGGTACKTKAAGCMAC A

Promoter 4

GCGRAAAAAA AAAACAGATA AAAGTAARTC AAACATATAT CCTGCWACWCGCGCGKATGT CGGTCATCRT
GTSYCGCCCM RKGTMCGYST SCWGTRCSTCCCTAGTGACT CACTAGGGGA CTTTCCCTAG GCACGTCTAG
GCACGTCTAGTGAGTCACTAGGCACGTCTAGCTCGCGATCTTATGATCTGGATCCATGCTCGAGGCCTGTAGG
CGTGTAC GGTGGGAGGC TTA**TATAA**GC AGAGCTCAAGCTGGCATCCG TACKTGAGCC AT

Promoter 6

GCATCACAAATTTCACAAATAAAGCATTTTTTTCACTGCATTCTAGTTGTGGTTTGTCCAAACTCATCAATGTAT
CTTATCATGTCTGGCCAGCTAGACGCGTGCTAGGGAAAGTCCCCTAGACGTGCGGAAAGTCCCCTAGGCACG
TCTAGTGAGTCAC TAGGGGACTTTCCCTAGTGACTCACTAGTGACTCACTAGCTCGCGATCTTATGATCTGGA
TCCATGCTCG AGGCCTGTAG GCGTGTACGGTGGGAGGCTT A**TATAA**GCAG AGCTCAAGCT GGCATCCGGT
ACKKGRAGCC

Promoter 7

TTATAATGGTTACAAATAAAGCAATAGCATCACAAATTTCACAAATAAAGCATTTTTTTCACTGCATTCTAGTTG
TGGTTTGTCCAAACTCATCAATGTATCTTATCATGTCTGGCCAGCTAGACGCGTGCTAGGGAAAGTCCCCTAG
ACGTGCCTAGGCACGTCTAGTGAGTCACTAGACGTGCCTAGACGTGCCTAGACGTGCCTAGCTCGCGATCTT
ATGATCTGGATCCATGCTCGAGGCCTGTAGGCGTGTACGGTGGGAGGCTTA**TATAA**GCAGAGCTCAAGCTTG
GCATCCGG

Promoter 8

WGRKTCCTSCCRCACCTCCCCSTGAACCTGAAACATAAAATGAATGCAATTGTTGTTGTTAACTTGTTTATTGCA
GCTTATAATGGTTACAAATAAAGCAATAGCATCACAAATTTCRCAAATAAAGCATTTTTTTCACTGCATTCTAGT
TGTGGTTTGTCCAAACTCATCAATGTATCTTATCATGTCTGGCCAGCTAGACGCGTGCTAGTGAGTCACTAGAC
GTGCCTAGGCACGTCTAGCTCGCGATCTTATGATCTGGATCCATGCTSGAGGCCTGTAGGCGTGTACGGTGG
GAGGCTTA**TATAA**GCAGAGCTCAAGCTKGCATCCGTACGTTKAGCCMMCCC

Promoter 9

TTATAATGGTTACAAATAAAGCAATAGCATCACAAATTTCACAAATAAAGCATTTTTTTCACTGCATTCTAGTTG
TGGTTTGTCCAAACTCATCAATGTATCTTATCATGTCTGGCCAGCTAGACGCGTGCTAGTGACTCACTAGGGA
AAGTCCCCTAGTGACTCACTAGACGTGCCTAGGCACGTCTAGTGACTCACTAGCTCGCGATCTTATGATCTGG
ATCCATGCTCGAGGCCTGTAGGCGTGTACGGTGGGAGGCTTA**TATAA**GCAGAGCTCAAAGCTGGCATCCGTA

Promoter 10

TTATAATGGTTACAAATAAAGCAATAGCATCACAAATTTCACAAATAAAGCATTTTTTTCACTGCATTCTAGTTG
TGGTTTGTCCAAACTCATCAATGTATCTTATCATGTCTGGCCAGCTAGACGCGTGCTAGGGAAAGTCCCCTAG
GCACGTCTAGGCACGTCTAGGGGACTTTCCCTAGTGACTCACTAGACGTGCCTAGACGTGCCTAGTGACTCA
CTAGGCACGTCTAGACGTGCCTAGGCACGTCTAGGCACGTCTAGGGGACTTTCCCTAGTGACTCACTAGGCA
CGTCTAGGCACGTCTAGTGACTCACTAGTGACTCACTAGGGGACTTTCCCTAGTGAGTCACTAGTGACTCAC
TAGGCACGTCTAGTGACTCACTAGCTCGCGATCTTATGATCTGGATCCATGCTCGAGGCCTGTAGGCGTGTAC
GGTGGGAGGCTTA**TATAA**GCAGAGNTCAAGCTGGCATTCCGTAC

Promoter 11

TTTCACAAATAAAGCATTTTTTTCACTGCATTCTAGTTGTGGTTTGTCCAAACTCATCAATGTATCTTATCATGTC
TGGCCAGCTAGACGCGTGCTAGGGGACTTTCCCTAGGCACGTCTAGTGACTCACTAGGGGACTTTCCCTAGA
CGTGCCTAGTGA GTCACTAGCT CGCGATCTTA TGATCTGGATCCATGCTCGAGGCCTGTAGGCGTGTACGGT
GGGAGGCTTA **TATAA**GCAGA GCTCAAGCTTGGCATCCGGT ACKTKKAGCC ACTA

Promoter 12

TTATAATGGTTACAAATAAAGCAATAGCATCACAAATTTCACAAATAAAGCATTTTTTTCACTGCATTCTAGTTG
TGGTTTGTCCAAACTCATCAATGTATCTTATCATGTCTGGCCAGCTAGACGCGTGCTAGTGACTCACTAGGGA
AAGTCCCCTAGACGTGCCTAGTGACTCACTAGGGGACTTTCCCTAGACGTGCCTAGACGTGCCTAGTGACTC
ACTAGTGAGTCACTAGGGAAAGTCCCCTAGCTCGCGATCTTATGATCTGGATCCATGCTCGAGGCCTGTAGG
CGTGTACGGTGGGAGGCTTA**TATAA**GCAGAGCTCAAGCTGGCATTCCGTA

Promoter 14

TACCAACAGTACCGGAATGCCAAGCTTGAGCTCTGCTTATATAAGCCTCCCACCGTACACGCCTACAGGCCTCG
AGCATGGATCCAGATCATAAGATCGCGAGCTAGTGACTCACTAGTGACTCACTAGTGAGTCACTAGTGAGTC
ACTAGCTCGCGATCTTATGATCTGGATCCATGCTCGAGGCCTGTAGGCGTGTACGGTGGGAGGCTTA**TATAA**
GC AGAGCTCAAG CTGGCATCCGGTACKKRASC MAGTAC

Promoter 15

AAACCTCCCACACCTCCCCCTGAACCTGAAACATAAAATGAATGCAATTGTTGTTGTTAACTTGTTTATTGCAGC
TTATAATGGTTACAAATAAAGCAATAGCATCACAAATTTCACAAATAAAGCATTTTTTTCACTGCATTCTAGTTG
TGGTTTGTCCAAACTCATCAATGTATCTTATCATGTCTGGCCAGCTAGACGCGTGCTAGGCACGTCTAGTGAG
TCACTAGGGGACTTTCCCTAGCTCGCGATCTTATGATCTGGATCCATGCTCGRGGCCTGTAGGCGTGTACGGT
GGAGGCTTA**TATA A**GCAGAGCTCAAGCTGGCATCCGGTACKTGAGCCACCC

Promoter 17

AAATAAAGCAATAGCATCACAAATTTCACAAATAAAGCATTTTTTTCACTGCATTCTAGTTGTGGTTTGTCCAAA
CTCATCAATGTATCTTATCATGTCTGGCCAGCTAGACGCGTGCTAGGGGACTTTCCCTAGGCACGTCTAGTGA
CTCACTAGGGGACTTTCCCTAGACGTGCCTAGTGAGTCACTAGCTCGCGATCTTATGATCTGGATCCATGCTC
GAGGCCTGTAGGCGTGTACGGTGGGAGGC

Promoter 19

AACCTCCCACACCTCCCCCTGAACCTGAAACATAAAATGAATGCAATTGTTGTTGTTAACTTGTTTATTGCAGCT
TATAATGGTTACAAATAAAGCAATAGCATCACAAATTTCACAAATAAAGCATTTTTTTCACTGCATTCTAGTTGT
GGTTTGTCCAAACTCATCAATGTATCTTATCATGTCTGGCCAGCTAGACGCGTGCTAGGCACGTCTAGGCACG
TCTAGCTCGCGATCTTATGATCTGGATCCATGCTCGAGGCCTGTAGGCGTGTACGGTGGGAGGCTTA**TATAA**
GCAGAGCTCAAGCTGGCATCCGGTACKTGAAGCCA CC

Assembly PCR Promoters

Promoter 1

ACSTCCCACACCTCCCCCTGAACCTGAAACATAAAATGAATGCAATTGTTGTTGTTAACTTGTTTATTGCAGCTT
ATAATGGTTACAAATAAAGCAATAGCATCACAAATTTCACAAATAAAGCATTTTTTTCACTGCATTCTAGTTGT
GGTTTGTCCAAACTCATCAATGTATCTTATCATGTCTGGCCAGCTAGCGTGCCTCTTATGATCTGGATGGGACT
TTCCATCTCTGCGATGAACCTCACTGAGTCAGTGCCTCTTATGATCTGGAT**CCATG**ATCTCTGCGATGAACCTC
ACTGAGTCATATGATCTGGATTGAGTCAATCTCTGCGATGAACCTCACGGGACTTTCCGTGCCTCTTATGATCT
GGATGGGACTTTCCATCTCTGCGATGAACCTCACTGAGTCAGTGCCTCTTATGATCTGGATTGAGTCAATCTC
GCGATGAACCTCACACGTGCGTGCCTCTTATGATCTGGATTGAGTCAATCTCTGCGATGAACCTCACACGTGC
GT GCCTCTTATG ATCTGGATACGTGCATCTCT GCGATGAACC TCACACGTGC GTGCCTCTTATGATCTGGAT
CTCGAGCCATGGTCGAGGCCTGTAGGCGTGTACGGTGGGAGGCTTA**TATA A**GCAGAGCTCAAGCTGGCAT

Promoter 2

CAGCTTATAATGGTTACAAATAAAGCAATAGCATCACAAATTTCACAAATAAAGCATTTTTTTCACTGCATTCTA GTTGTGGTTTGTCCAAACTCATCAATGTATCTTATCATGTCTGGCCAGCTAGCGTGCCTCTTATGATCTGGATT GAGTCAATCTC TGCGATGAAC CTCACTGAGT CAGTGCCTCT TATGATCTGGATACGTGCAT CTCTGCCGAT GAACCTCACTGAGTCAGTGCCTCTTATGATCTGGATACGTGCATCTCTGCGATGAACCTCACTGAGTCAGTGC CTCTTATGATCTGGATTGAGTCAATCTCTGCGATGAACCTCACTGAGTCAGTGCCTCTTATGATCTGGATTGAG TCA ATCTCTGCGA TGAACCTCACACGTGCGTGCCTCTTATGATCTGGATGGGACTTTCCATCTCTGCGATGAA CCTCACGGGACTTTCCGTGCCTCTTATGATCTGGATGGGACTTTCCATCTCTGCGATGAACCTCACTGAGTCAG TGCCTCTTATGATCTGGATCTCGAGCCATGGTCGAGGCCTGTAGGCGTGTACGGTGGGAGGCTTA**TATAA**GC AGAG CTCAAGCTTGGCATCCGGTA CKKGAGCCMM CC

Promoter 3

CATGTCTGGCCAGCTAGCGTGCCTCTTATGATCTGGATACGTGCATCTCTGCGATGAACCTCACGGGACTTTCC GTGCCTCTTATGATCTGGATACGTGCATCTCTGCGATGAACCTCACACGTGCGTGCCTCTTATGATCTGGATTG AGTCAATCTCTGCGATGAACCTCACGGGACTTTCCGTGCCTCTTATGATCTGGATACGTGCATCTCTGCGATG AACCTCACTGAGTCAGTGCCTCTTATGATCTGGATACGTGCATCTCTGCGATGAACCTCACACGTGCGTGCCT CTTATGATCTGGATCTCGAGCCATGGTCGAGGCCTGTAGGCGTGTACGGTGGGAGGCTTA**TATAA**GCAGAGC TCAA GCTGGCATCC GGTACKTGAG CACTTT

Promoter 4

CATTCTAGTTGTGGTTTGTCCAAACTCATCAATGTATCTTATCATGTCTGGCCAGCTAGCGTGCCTCTTATGATC TGGATTGAGTCAATCTCTGCGATGAACCTCACTGAGTCAGTGCCTCTTATGATCTGGATACGTGCATCTCTGC GATGAACCTCACACGTGCGTGCCTCTTATGATCTGGATGGGACTTTCCATCTCTGCGATGAACCTCACACGTG CGTGCCTCTTATGATCTGGATGGGACTTTCCATCTCTGCGATGAACCTCACGGGACTTTCCGTGCCTCTTATGA TCTGGATGGGACTTTCCATCTCTGCGATGAACCTCACTGAGTCAGTGCCTCTTATGATCTGGATGGGACTTTCC ATCTCTGCGATGAACCTCACGGGACTTTCCGTGCCTCTTATGATCTGGATACGTGCATCTCTGCGATGAACCTC ACTGAGTCAGTGCCTCTTATGATCTGGATACGTGCATCTCTGCGATGAACCTCACTGAGTCAGTGCCTCTTATG ATCTGGATCTCGAGCCATGGTCGAGGCCTGTAGGCGTGTACGGTGGGAGGCTTA**TATAA**GCAGAGCTCAAGC T TGGCATCCGG

Promoter 5

GCAATAGCGTCACAAATTTCACAAATAAAGCATTTTTTTCACTKCRTTSTAGTTGWGGTTTGTCCAAACTCATCA ATGTATCTTATCATGTCTGGCCAGCTAGCGTGCCTCTTATGATCTGGATACGTGCATCTCTGCGATGAACCTCA CACGTGCGTGCCTCTTATGATCTGGATACGTGCATCTCTGCGATGAACCTCACTGAGTCAGTGCCTCTTATGAT CTGGATCTCGAGCCAWGGKSGAGGCCTGTAGGCGTGTACGGTGGGAGGCTTA**TATAA**GCAGAGCTCAAGCT TGGCATCCGGTACTGT GARCCWYCTT

Promoter 6

AAACTCATCAATGTATCTTATCATGTCTGGCCAGCTAGCGTGCCTMTTATRATCTGGAKTGAGTCAATCTCTGC
GATGAACCTCACACGTGCGTGCCTCTTATGATCTGGATACGTGCATCTTTGCGATGAACCTCACGGGACTTTC
CGTGCCTCTTATGATCTGGATACGTGCATCTCTGCGATGAACCTCACACGTGCGTGCCTCTTATGATCTGGAT
GGGACTTTCCATCTCTGCGATGAACCTCACACGTGCGTGCCTCTTATGATCTGGATGGGACTTTCCATCTCTGC
GATGAACCTCACACGTGCGTGCCTCTTATGATCTGGATTGAGTCAATCTCTGCGATGARCSTCACACGTGCGT
GCCTCTTATGATCTGGATCTCGAGCCATGGTSGAGGCCTGTAGGCGTGTACGGTGGGAGGCTTA**TATAA**GCA
GAGCTCAAGCTGGCATTCCGGTAYKKGKYRRCCKCCTTYTG

Promoter 9

TTCTAGTTGTGGTTTGTCCAAACTCATCAATGTATCTTATCATGTCTGGCCAGCTAGCGTGCCTCTTATGATCTG
GATTGAGTCAATCTCTGCGATGAACCTCACACGTGCGTGCCTCTTATGATCTGGATGGGACTTTCCACCTCTGC
GATGAACCTCACACGTGCGTGCCTCTTATGATCTGGATGGGACTTTCCATCTCTGCGATGAACCTCACTGAGT
CAGTGCCTCTTATGATCTGGATTGAGTCAATCTCTGCGATGAACCTCACGGGACTTTCCGTGCCTCTTATGATC
TGGATACGTGCATCTCTGCGATGAACCTCACTGAGTCAGTGCCTCTTATGATCTGGATGGGACTTTCCATCTCT
GCGATGAACCTCACACGTGCGTGCCTCTTATGATCTGGATTGAGTCAATCTCTGCGATGAACCTCACACGTGC
GTGCCTCTTATGATCTGGATCTCGAGCCATGGTCGAGGCCTGTAGGCGTGTACGGTGGGAGGCTTA**TATAA**G
CAGAGCTCAAGCTGGCATCCGGTACKTGAGMCMCC

Promoter 11

GGTTTGTCCAAACTCATCAATGTATCTTATCATGTCTGGCCAGCTAGCGTGCCTCTTATGATCTGGATGGGACT
TTCCATCTCTGCGATGAACCTCACTGAGTCAGTGCCTCTTATGATCTGGATTGAGTCAATCTCTGCGATGAACC
TCACACGTGCGTGCCTCTTATGATCTGGATACGTGCATCTCTGCGATGAACCTCACGGGACTTTCCGTGCCTCT
TATGATCTGGATGGGACTTTCCATCTCTGCGATGAACCTCACTGAGTCAGTGCCTCTTATGATCTGGATCTCGA
GCCATGGTCGAGGCCTGTAGGCGTGTACGGTGGGAGGCTTA**TATAA**GCAGAGCTCAAGCTGGCATCCGGTAC
KK RAGRCCCC

Promoter 12

CCAGCTAGCGTGCCTCTTATGATCTGGATTGAGTCAATCTCTGCGATGAACCTCAGGGGACTTTCCGTGCCTCT
TATGATCTGGATGGGACTTTCCATCTCTGCGATGAACCTCACTGAGTCAGTGCCTCTTATGATCTGGATTGAGT
CAATCTCTGCGA TGAACCTCAC GGGACTTTCC GTGCCTCTTA TGATCTGGATACGTGCATCTCTGCGATGAA
CCTCACGGGACTTTCCGTGCCTCTTATGATCTGGATCTCGAGCCATGGTCGAGGCCTGTAGGCGTGTACGGTG
GGAGGCTTATATAAGCAGAGCTCAAGCTGGCATCCGGTACKTGAGACMCCCTC

Promoter 13

RACSTCCCRCACCTCCCCCTGAACCTGAAACATAAAATGAATGCAATTGTTGTTGTTAACTTGTTTATTGCAGCT
TATAATGGTTACAAATAAAGCAATAGCATCACAAATTTCACAAATAAAGCATTTTTTTCACTGCATTCTAGTTGT
GGTTTGTCCAAACTCATCAATGTATCTTATCATGTCTGGCCAGCTAGCGTGCCTCTTATGATCTGGATACGTGC
ATCTCTGCGATGAACCTCACTGAGTCAGTGCCTCTTATGATCTGGATACGTGCATCTCTGCGATGAACCTCACT
GAGTCAGTGCCTCTTATGATCTGGATTGAGTCAATCTCTGCGATGAACCTCACTGAGTCAGTGCCTCTTATGAT
CTGGATGGGACTTTCCATCTCTGCGATGAACCTCACGGGACTTTCCGTGCCTCTTATGATCTGGATTGAGTCA
ATCTCTGCGATGAACCTCACACGTGCGTGCCTCTTATGATCTGGATTGAGTCAATCTCTGCGATGAACCTCAC
GGGACTTTCCGTGCCTCTTATGATCTGGATCTCGAGCCATGGTCGAGGCCTGTAGGCGTGTACGGTGGGAGG
CTTA**TATAA**GCAGAGCTCAA GCTGGCATCC GTACGTGARC CACCTC

Promoter 14

AAACTCATCAATGTATCTTATCATGTCTGGCCAGCTAGCGTGCCTCTTATGATCTGGATGGGACTTTCCATCTCT
GCGATGAACCTCACGGGACTTTCCGTGCCTCTTATGATCTGGATTGAGTCAATCTCTGCGATGAACCTCACGG
GACTTTCCGTGCCTCTTATGATCTGGATACGTGCATCTCTGCGATGAACCTCACACGTGCGTGCCTCTTATGAT
CTGGATGGGACTTTCCATCTCTGCGATGAACCTCACACGTGCGTGCCTCTTATGATCTGGATCTCGAGCCATG
GTCGAGGCCTGTAGGCGTGTACGGTGGGAGGCTTA**TATAA**GCAGAGCTCAAGCTGGCATCCGGTACKTKRAA
RCCCMCCC

Promoter 15

ATTTCACAAATAAAGCATTTTTTTCACTGCATTCTAGTTGTGGTTTGTCCAAACTCATCAATGTATCTTATCATGT
CTGGCCAGCTAGCGTGCCTCTTATGATCTGGATGGGACTTTCCATCTCTGCGATGAACCTCACGGGACTTTCC
GTGCCTCTTATGATCTGGATCTCGAGCCATGGKCGAGGCCTGTAGGCGTGTACGGTGGGAGGCTTA**TATAA**G
CAGAGCTCAAGCTGGCATCCGGTACTKTG

Promoter 16

GTGTCYKGGCCCAGCTAGCGTGCCTCTTATGATYTGGGATTGAGTCAATCTCTGCGATGAACCTCACGGGRAC
TTCCGTGCCTCTTATGATCTGGGATACGTGCATCTCTGCGATGAACCTCACTGAGTCAGTGCCTCTTTATGATC
TGGATACGTGCATCTSTGCGATGAACCTCACACGTGCGTGCCTCTTATGATCTGGATTGAGTCAATCTCTGCG
ATGAACCTCACTGAGTCAGTGCCTCTTATGATCTGGATTGAGTCAATCTCTGCGATGAACCTCACGGGACTTT
CCGTGCCTCTTATGATCTGGATGGGACTTTCCATCTCTGCGACCTCACTGAGTCAGTGCCTCTTATGATCTGGA
TGGGACTTTCCATCTCTGCGATGAACCTCACACGTGCGTGCCTCTTATGATCTGGATGGGACTTTCCATCTCTG
CGATGAACCTCACGGGACTTTCCGTGCCTCTTATGATCTGGATGGGACTTTCCATCTCTGCGATGAACCTCACT
GAGTCAGTGCCTCTTATGATCTGGATACGTGCATCTCTGCGATGAACCTCACACGTGCGTGCCTCTTATGATCT
GGATACGTGCATCTCTGCGATGAACCTCACGGGACTTTCCGTGCTCTTATGATCTGGATACGTGCATCTCTGC
GATGAACCTCACACGTGCGTGCCTCTTATGATCTGGAT**GGGC**ATCTCTGCGATGAACCTCACACGTGCGTGCC
TCTTATGATCTGGATCTCGAGCCATGGTCGAGGCCTGTAGGCGTGTACGGTGGGAGGCTTA**TATAA**GCAGAG
CTC AAGCTTGGCA TCCGGTACCK TGAGCMCCC

Promoter 17

GAGATCYTSYCACACCTCCCCCTGARCYTGAAACATAAAATGAATGCAATTGTTGTTGTTAACTTRTTTATTGCA
GCTTATAATGGTTACAAATAAAGCAATAGCATCACAAATTTCACAAATAAAGCATTTTTTTCACTGCATTCTAGT
TGTGGTTTGTCCAAACTCATCAATGTATCTTATCATGTCTGGCCAGCTAGCGTGCCTCTTATGATCTGGATACG
TGCATCTCTGCGATGAACCTCACTGAGTCAGTGCCTCTTATGATCTGGATACGTGCATCTCTGCGATGAACCTC
ACTGAGTCAGTGCCTCTTATGATCTGGATTGAGTCAATCTCTGCGATGAACCTCACGGGACTTTCCGTGCCTCT
TATGATCTGGATCTCGAGCCATGGTSGAGGCCTGTAGGCGTGTACGGTGGGAGGCTTA**TATAA**GCAGAGCTA
AGCTTGGCATCCGTACYGTTGAGRCMMCCCCA

Promoter 18

TGTATCTTATCATGTCTGGCCAGSTAGCGTGCCTCTTATGATCTGGATGGGACTTTCCATCTCTGCGATGAACC
TCACACGTGCGTGCCTCTTATGATCTGGATGGGACTTTCCATCTCTGCGATGAACCTCACGGGACTTTCCGTGC
CTCTTATGATCTGGATTGAGTCAATCTCTGCGATGAACCTCACGGGACTTTCCGTGCCTCTTATGATCTGGATG
GGACTTTCCATCTCTGCGATGAACCTCACACGTGCGTGCCTCTTATGATCTGGATCTCGAGCCATGGTCGAGG
CCTGTAGGCGTGTACGGTGGGAGGCTTA**TATAA**GCAGAGCTCAAGCTGGCATCCGGTACKTTAGCACTGGCA

Promoter 19

TGTCCAAACTCATCAATGTATCTTATCATGTCTGGCCAGCTAGCGTGCCTCTTATGATCTGGATTGAGTCAATCT
CTGCGATGAACCTCACGGGACTTTCCGTGCCTCTTATGATCTGGATACGTGCATCTCTGCGATGAACCTCACT
GAGTCAGTGCCTCTTATGATCTGGATGGGACTTTCCATCTCTGCGATGAACCTCACTGAGTCAGTGCCTCTTAT
GATCTGGATGGGACTTTCCATCTCTGCGATGAACCTCACACGTGCGTGCCTCTTATGATCTGGATGGGACTTT
CCATCTCTGCGATGAACCTCACTGAGTCAGTGCCTCTTATGATCTGGATACGTGCATCTCTGCGATGAACCTCA
CACGTGCGTGCCTCTTATGATCTGGATCTCGAGCCATGGTCGAGGCCTGTAGGCGTGTACGGTGGGAGGCTT
A**TATAA**GCAGAGCTCAAGCTGGCATCCGGTACKTGAGCA CTTG

Promoter 20

GTATCTTATCATGTCTGGCCAGCTAGCGTGCCTCTTATGATCTGGATTGAGTCAATCTCTGCGATGAACCTCAC
TGAGTCAGTGCCTCTTATGATCTGGATGGGACTTTCCATCTCTGCGATGAACCTCACACGTGCGTGCCTCTTAT
GATCTGGATACGTGCATCTCTGCGATGAACCTCACTGAGTCAGTGCCTCTTATGATCTGGATCTCGAGCCAWG
RTSGAGGCCTGTAGGCGTGTACGGTGGGARGCTTA**TATAA**GCAGAGCTCAAGCTTKGCWTTCCGKAAYCTRYS
GTTKKA
